# Spatially constrained tumour growth affects the patterns of clonal selection and neutral drift in cancer genomic data

**DOI:** 10.1371/journal.pcbi.1007243

**Published:** 2019-07-29

**Authors:** Ketevan Chkhaidze, Timon Heide, Benjamin Werner, Marc J. Williams, Weini Huang, Giulio Caravagna, Trevor A. Graham, Andrea Sottoriva

**Affiliations:** 1 Evolutionary Genomics and Modelling Lab, Centre for Evolution and Cancer, The Institute of Cancer Research, London, United Kingdom; 2 Evolution and Cancer Lab, Barts Cancer Institute, Queen Mary University of London, London, United Kingdom; Stanford University School of Medicine, UNITED STATES

## Abstract

Quantification of the effect of spatial tumour sampling on the patterns of mutations detected in next-generation sequencing data is largely lacking. Here we use a spatial stochastic cellular automaton model of tumour growth that accounts for somatic mutations, selection, drift and spatial constraints, to simulate multi-region sequencing data derived from spatial sampling of a neoplasm. We show that the spatial structure of a solid cancer has a major impact on the detection of clonal selection and genetic drift from both bulk and single-cell sequencing data. Our results indicate that spatial constrains can introduce significant sampling biases when performing multi-region bulk sampling and that such bias becomes a major confounding factor for the measurement of the evolutionary dynamics of human tumours. We also propose a statistical inference framework that incorporates spatial effects within a growing tumour and so represents a further step forwards in the inference of evolutionary dynamics from genomic data. Our analysis shows that measuring cancer evolution using next-generation sequencing while accounting for the numerous confounding factors remains challenging. However, mechanistic model-based approaches have the potential to capture the sources of noise and better interpret the data.

## Author summary

Sequencing the DNA of cancer cells from human tumours has become one of the main tools to study cancer biology. However, sequencing data are complex and often difficult to interpret. In particular, the way in which the tissue is sampled and the data are collected impact the interpretation of the results significantly. We argue that understanding cancer genomic data requires mechanistic mathematical and computational models that tell us what we expect the data to look like, with the aim of understanding the impact of confounding factors and biases in the data generation step. In this study, we develop a spatial computational model of tumour growth that also simulates the data generation process, and demonstrate that biases in the sampling step and current technological limitations severely impact the interpretation of the results. We then provide a statistical framework that can be used to start overcoming these biases and more robustly measure aspects of the biology of tumours from the data.

## Introduction

Cancer is an evolutionary process fuelled by genomic instability and intra-tumour heterogeneity (ITH) [1]. ITH leads to therapy resistance, arguably the biggest problem in cancer treatment today [2]. Recently, seminal studies have attempted to quantify ITH by either looking at subclonal mutations in deep sequencing data from single bulk samples [3,4], or by taking multiple samples of the same tumour, the so-called multi-region sequencing approach (reviewed in [5]). Phylogenetic approaches are then used to reconstruct the ancestral history of cancer cell lineages [6]. However, one important difference between phylogenetic analyses in cancer and classical phylogenetic analyses of species is that each cancer sample is not a single individual, but a mixture of different cancer cell subpopulations and non-cancer cells [7].

The problem is usually tackled by performing subclonal deconvolution of the samples to separate the different subpopulations [3,8]. However, these approaches do not account for the spatio-temporal dynamics that generated the data. To study the evolutionary dynamics of individual tumours, mathematical and computational models of evolutionary processes are widely employed [9–12]. Many of these models are rooted in theoretical population genetics, a field that quantifies the evolution of alleles in populations and that is central to the modern evolutionary synthesis [13]. More recently, spatial models have also been used [14–23]. However, seldom have mathematical and computational models of cancer evolution been directly connected to next-generation sequencing data from human tumours. Recent work from us and others has shown that combining theoretical modelling and cancer genomic data allows for measurement of fundamental properties of the tumour evolutionary process *in vivo*, such as mutation rates and strength and onset of subclonal selection events [22,24,25].

Here, we study how spatial constrains of a growing tumour impact our ability to infer cancer evolutionary dynamics. We combine explicit spatial evolutionary modelling with synthetic generation of multi-region bulk and single-cell data, thus providing a generative framework in which we know the evolutionary trajectories of all cells in a tumour and can examine the genomic patterns that emerge from the sampling experiment. We show that spatial constrains, stochastic spatial growth and sampling biases can have unexpected effects that confound both the interpretation and inference of the perceived evolutionary dynamics from cancer sequencing data. We also present a statistical inference framework that begins to account for some of these confounding factors and recover aspects of the cancer evolutionary dynamics from various types of multi-region sequencing data as well as single-cell data.

## Results

### Simulating spatial tumour growth, sampling and data generation

**Fig 1:**
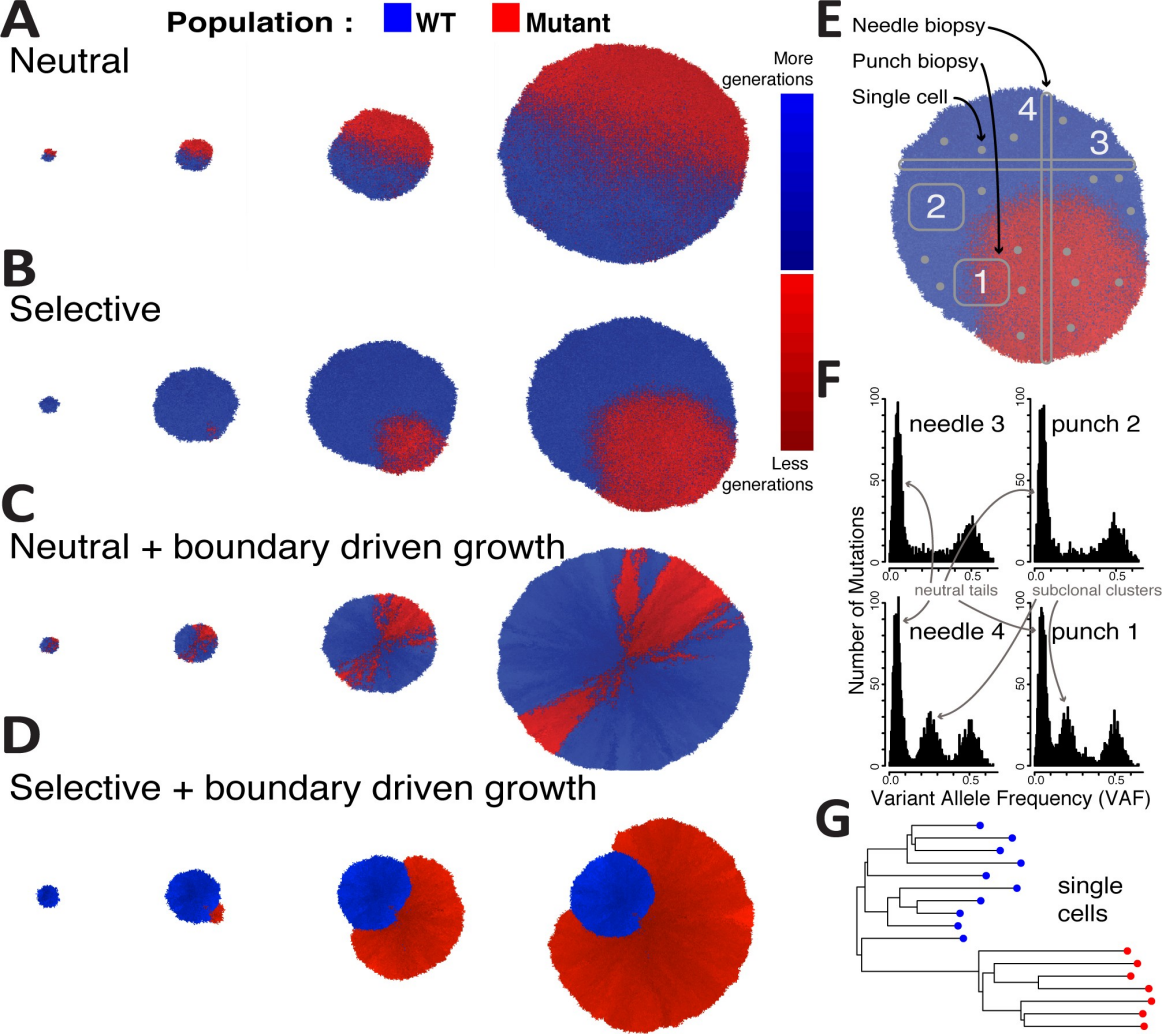
A spatial tumour growth model that simulates sequencing data. In our model we introduce a mutant at a given time t (blue = background clone; red = mutant subclone; shade is proportional to the number of generations the cell has gone through). **(A)** The new mutant subclone can have no fitness advantage (mutation is a passenger), giving rise to a neutrally growing neoplasm, or **(B)** have a fitness advantage s>0 with respect to the background population (mutation is a driver), giving rise to differential selection in the tumour population. In addition, cells accumulate unique passenger mutations during each cell division. **(C)** In some tumours, it is likely that only cells close to the tumour border are able to proliferate due to the abundance of resources and space. We simulate this in our model as boundary driven growth, which gives rise to complex radial patterns. **(D)** When boundary driven growth is combined with selection, spatial effect can either amplify the growth of the new subclone, as in this exemplary case, or even decrease the effects of selection if the subclone, by chance, gets imprisoned behind the growing front. **(E)** In our simulation we also model the raising and spread of point mutations in the genome of cancer cells (all passengers and, when subclone is selective, one additional driver). We can simulate the sampling of punch biopsies (squares), needle biopsies (thin stripes) and single cells. **(F)** By simulating the noise and measurement errors of next-generation sequencing, we can generate synthetic realistic variant allele frequency distributions from the spatial simulations. **(G)** Single-cell data can also be simulated, in this case clearly showing the presence of a selected subclone demonstrated by the clade of “red” cells with a recent common ancestor.

Here we develop and analyse a stochastic spatial cellular automaton model of tumour growth that incorporates cell division, cell death, random mutations and clonal selection (Material and Methods). Each tumour simulation starts with a single ‘transformed’ cell in the centre of either a 2D or a 3D lattice, and we model the resulting expansion of this first cancer cell. All events, such as cell proliferation, death, mutation and selection are modelled according to a Gillespie algorithm [26]. In our model we account for different spatial constraints that are parameterised within our simulation. In order for a cell to divide, a new empty space for its progeny is required within the 8 neighbouring cells if we consider a 2D grid with Von Neumann neighbourhood. If no empty space is present, a cell can generate a new space by pushing neighbouring cells outwards (choosing a random direction of the push). In this scenario, the growth is ‘homogeneous’ and all cells in the neoplasm can divide (Fig 1A and 1B). Because all cells in the tumour can divide, this scenario leads to an overall exponential expansion (S1A and S1B Fig). At some point during the simulation (Fig 1A–1D), within the original tumour population (blue cells), we introduce a new mutant (a new subclone–red cells) which may or may not have a selective advantage. In the case of a neutral subclone (no selective advantage), the mutant cells proliferate as all the other cells (Fig 1A). We note that in this case, colouring a new subclone in red at a certain point during neutral growth is arbitrary, and equivalent to the marking of a lineage by a random neutral (passenger) mutation. In the case where the subclone has a fitness advantage, the mutant will, on average, grow more rapidly compared to the parental background clone, thus increasing in relative proportion over time (Fig 1B and S1B Fig).

We also model ‘boundary driven’ growth, where only cells that are sufficiently close to the border of the tumour can proliferate. Other cells may remain ‘imprisoned’ in the centre of the tumour unable to proliferate because of the lack of empty space around them. Boundary-driven growth has been observed experimentally [27–29] as well as in model systems [30]. The magnitude of this effect is controlled in our simulation with the parameter *a*, which considers cell location and defines the probability that a cell will push neighbouring cells to create empty spots depending on how far is the cell from the boundary (see Materials and Methods). Boundary driven growth leads to a polynomial expansion (S1C Fig). Importantly, in both the case of neutral mutants (Fig 1C) and selected mutants (Fig 1D), the spatial distribution of mutant cells in this scenario is strongly affected by the spatial constraints.

At each division, a cell has a certain probability to acquire additional somatic mutations, modelled with a Poisson distribution, with mean *u*, in line with many other previous models [11,24,25,31,32]. Notably, *u* is the average number of new somatic mutations per division for the whole genome of a single cell. We assume that both daughter cells can acquire mutations, that mutations are unique (infinite site model) and we neglect back mutations (infinite allele model). Finally, the large majority of mutations are assumed to be passengers (neutral), with a few driver alterations allowing for subclonal fitness advantages (e.g. subclonal populations in Fig 1B and 1D). This is consistent with large-scale genomic sequencing studies indicating that in any given tumour, the number of driver events is generally small, while the number of passengers is often orders of magnitude larger [31,33].

Importantly, our spatial model of tumour growth allows for the simulation of tissue sampling and genomic data generation. For instance, we can simulate the collection of punch biopsies, where spatially localised chunks of tumour are collected (Fig 1E). We can also simulate needle biopsies, where a long and thin piece of tissue is sampled (Fig 1E). We can then simulate the genomic data generation process starting from the cells in the sample and the identification of somatic mutations. For example, we can simulate the sequencing at a given coverage using Binomial sampling of the alleles, the limits of low frequency mutation detection (e.g. minimum number of reads with a variant, minimum coverage), as well as non-uniformity of coverage leading to over-dispersion of the variant allele frequency (VAF) of detected mutations. This allows generating realistic data from simulated tumours, e.g. in the case of the simulation of a diploid tumour with one selected subclone in Fig 1E, all needles and punch biopsies contained clonal mutations, shown as a cluster of variants around VAF = 0.5 (Fig 1F), and in the case of punch biopsy 1 and needle biopsy 4, also a subclonal cluster representing the growing subclone.

We previously showed, using a non-spatial stochastic branching process model of tumour growth, that assuming a well-mixed population and exponential growth, the expected VAF distribution of subclonal mutations in cancer under neutral growth follows a power-law with a *1/f*^*2*^ scaling behaviour, where *f* is the variant allele frequency of subclonal mutations [24]. This has been previously demonstrated to be the scaling solution of the fully stochastic Luria-Delbruck model [34–36]. The 1/f_2_-like neutral subclonal tail can be observed in all samples of Fig 1F. In the presence of subclonal selection, we expect to observe an additional subclonal ‘cluster’ of mutations all at the same frequency [3], that are the passenger mutations hitchhiking in the expanding clone (as we previously described [25]). This is exemplified in needle 4 and punch 1 in Fig 1F. We note that a 1/f_2_-like tail remains in the VAF frequency spectrum of all samples, as a consequence of within-clone neutral dynamics that remain on-going throughout the tumour’s growth [25]. Furthermore, our framework allows simulating single-cell data. For example, from the simulated tumour in Fig 1B we sample individual cells at random and simulate single-cell whole-genome sequencing (Fig 1G).

### Spatial effects on bulk sequencing data

**Fig 2:**
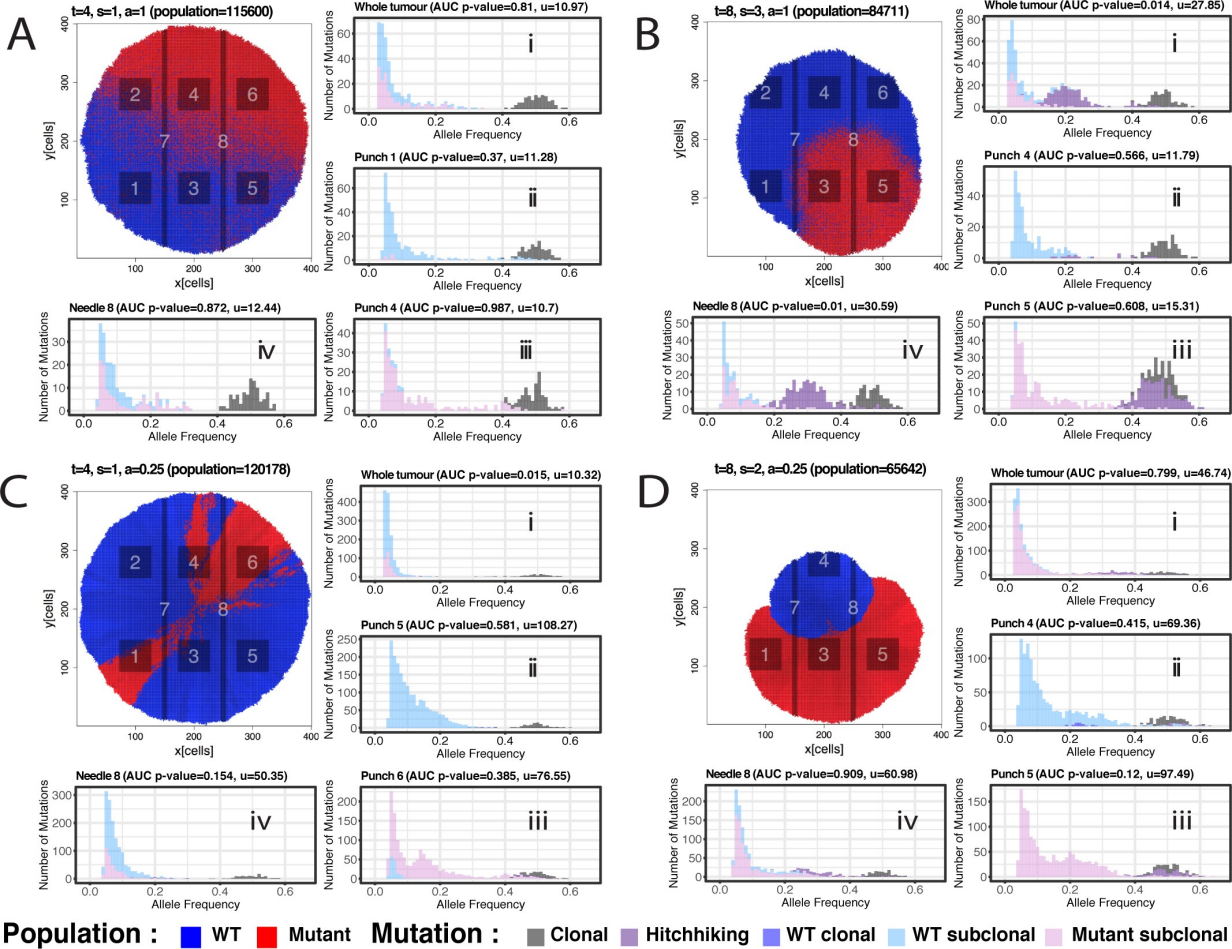
Variant allele frequency distributions of punch and needle biopsies from representative scenarios. **(A)** In the illustrative example of neutral homogeneous growth, a neutral mutant was introduced at generation time t = 4 with a selection coefficient of s = 0 (neutral) and homogeneous growth (a = 1). The mutation rate was u = 10. Tumour was simulated until ~100K cells. From the final tumour, we sampled 6 punch biopsies (1–6), 2 needle biopsies (7–8) and a “whole-tumour” sample, and simulated 100× whole-genome sequencing data. VAF distributions of each sample are shown (i-iv). **(B)** In this case, a differentially selected subclone with s = 3 was introduced at time t = 8 in a homogeneous growth scenario (a = 1) and u = 10. Final population size was ~80K cells. In those samples where both the background and the mutant subclone were present (i and iv), the VAF distribution showed evidence of subclonal selection, with a subclonal cluster (purple) generated by mutations in the selected subclone that hitchhiked to high frequency due to selection. **(C)** In the case of neutral boundary driven growth, a new (neutral) mutant was introduced at t = 4 with s = 1 and boundary driven growth parameter a = 0.025. Even though the tumour grew neutrally, the spatial effects of boundary driven growth led to deviations from the neutral expected null under homogeneous growth. Moreover, clusters in the VAF spectrum are detectable in iii, where sampling bias produced an over-representation of a lineage that was not due to selection. **(D)** Boundary driven growth with selection (mutant introduced at t = 8 with s = 2 and a = 0.025) produced even more complex patterns of drift and sampling bias. The data represents tumour simulations in 2D space. Birth rate b is 1 in all simulation.

For each representative simulation of spatial constraints in Fig 1, we modelled the sampling of 6 punch biopsies (small square regions), 2 needle biopsies (long and thin regions), as well as hypothetically sampling the whole tumour. From each sample, we simulated the generation of 100x depth whole-genome data (see Materials and Methods for details about the sequencing noise model). Fig 2A shows the variant allele frequency (VAF) distributions of samples from the neutral homogeneous growth case in Fig 1A, with clonal mutations (truncal) in grey, subclonal mutations exclusive to the parental background clone in light blue and subclonal mutations within the mutant in pink. All samples show the characteristic 1/f^2^ distribution corresponding to neutral evolutionary dynamics [24], as one would expect theoretically [34]. The Area Under the Curve (AUC) test for neutrality we previously proposed [25] (p<0.05 means neutrality is rejected) is reported on top of each VAF plot and shows that even in the presence of a spatial structure, homogeneous (exponential) neutral growth follows a 1/f^2^ distribution (Fig 2A-i to 2A-iv). As we have shown previously, it is possible to recover the mutation rate per cell doubling from the ~1/f^2^ neutral tail, which in this case without cell death was 10 mutations per division (~10^−9^ mutations/bp/division). This was correctly recovered in all samples from Fig 2A (recovered mutation rate reported in each plot as *u*).

In the case of homogeneous growth with subclonal selection (Fig 2B), neutrality could be rejected based in all those samples containing a mix of the background clone and the new subclone (Fig 2B-i and 2B-iv, see subclonal cluster in purple). Specifically, needle 4 and punch 1 showed the expected signature of selection, with a subclonal cluster a consequence of the over-representation of passenger mutations in the expanded clone [3,25]. The 1/f^2^-like tail resulting from the within-clone accumulation of passenger mutations remains in the frequency spectrum [25]. Specifically, in the plots in Fig 2B we report the mutations that were present in the first subclone cell in purple. Those are mutations that increased in frequency by hitchhiking on the selected mutant. Importantly, we note that these mutations are not exclusive to the subclone but are also found in other lineages (e.g. in the ‘cousins’ of the selected subclone). The same dynamics are observed if it is the death rate to decrease, rather than the birth rate to increase (S2A and S2B Fig). Importantly, the cell death *d* not only increases the rate of genetic drift, as expected, but also the level of clonal intermixing due to the additional stochasticity introduced by high cell replacement (S2C–S2F Fig, examples of neutral cases). Selection could not be detected in other spatially-distinct samples from the same tumour when they did not contain differentially selected populations, and either captured only the background clone (blue) or only the selected mutant (red) (Fig 2B-ii and 2B-ii). This is correct as in those samples ITH is neutral.

This initial spatial analysis produced similar results to our previous well-mixed non-spatial models [24,25]. We next investigated the effect of boundary driven growth. Here, only cells close to the borders grow, leaving other cells ‘imprisoned’ inside the tumour mass (see Materials and Methods for details), a pattern called *gene surfing* that causes radial patterns of cells growing only at the front of the expanding wave (Fig 2C). This has been previously documented both theoretically and experimentally in bacteria [37], in mathematical models of tumour growth [16,17,38], as well as in cancer model systems, where the neutral expansion of the cancer cell population under boundary driven growth led to lineages growing just because they were ‘lucky’ to be in the right place at the right time [29]. This has implications for the impact of the immune system during the evolution of a tumour, which exert a negative selection pressure on the cancer cell population through neoantigen recognition and removal [39], especially because neoantigen recognition is clone size dependent [40]. Importantly, boundary driven growth leads to non-exponential population dynamics [27,28] that also impact the distribution of mutations between the centre and the periphery of a solid neoplasm, as shown in a case of liver cancer sampled at high resolution [41]. The accumulation of subclonal mutations in a neutrally expanding tumour under boundary driven growth is expected to follow a 1/f^2^ scaling form within most of the detectable frequency range (f>5%), although at low frequency deviations are expected [37]. This is largely driven by the increasing difference in mutational burden between the centre and the border of the tumour, which could lead to rejection of the standard neutral expectation under exponential growth, as seen when the whole tumour is sampled with respect to when only a localised bulk/needle biopsy is collected (Fig 2C).

Because the population is no longer homogeneously distributed however, this can lead to significant spatial bias, causing over- or under-representation of mutations in the VAF distributions solely due to spatial effects and not because of selection. This causes deviations from the neutral expectation of the mutant allele distributions that risk being wrongly interpreted as the consequence of on-going subclonal selection, as in Fig 2C. In this scenario, we know that subclonal clusters (e.g. punch 6 in Fig 2C-iii) are not differentially selected subclones, but the over-representation of alleles is solely induced by the spatial structure. Furthermore, even when we observe distributions that appear to follow the neutral expectation (AUC p>0.05), boundary driven growth results in much higher mutational loads than would be expected in the well mixed case. Here our inferred mutation rates are up to 10 times higher than the ground truth. This can be observed more explicitly in S3 Fig, where we sample each representative tumour from the centre towards the periphery by taking samples along concentric circles (S3A Fig) and compare the mutational loads of the samples (S3B Fig). This was indeed observed in a case of neutrally growing liver cancer [41] and a similar phenomenon is also observed in species evolution [42].

If we combine boundary driven growth and subclonal selection the situation is further complicated: selective effects are now modulated by spatial constraints. In some cases, the selected mutant emerges and remains directly at the front of tumour growth. In this scenario the outgrowth caused by its selective advantage is amplified further just because it occurred at the growing front (Fig 2D). In other cases, the selected mutant may, by chance, remain ‘imprisoned’ within the tumour (assuming the mechanism of selective advantage is unable to overcome this spatial entrapment) and stops proliferating despite its selective advantage (e.g. S4 Fig). In both these cases, further sampling biases occur. In the case of punch 5 for example (Fig 2D-iii), where the new subclone is fixed (clone fraction = 100%), there is an overrepresentation of a cluster of mutations that is only due to spatial drift and not selection. These dynamics are recapitulated in larger cohorts of simulated tumours with the same parameters (S5 Fig). The distributions of p-values for the AUC measurements for all simulations for different modes of growth are illustrated in S6A Fig. This figure shows that neutrality is accepted in the majority of homogeneous cases without selection, and it is rejected in the majority of homogeneous cases with selection. In the case of boundary driven growth things are more complicated. In S6B Fig we show the AUC tests for neutrality applied to whole-tumour samples versus punch/needle biopsies. In the case of neutral boundary driven growth, neutrality is accepted in the majority of cases when we use localised punch/needle biopsies, but rejected when the whole-tumour sample is examined. This is due to the deviation from strict neutrality caused by boundary driven growth, that can be detected only when a large region of the tumour is sampled (and hence differences between centre and periphery of the tumour are captured). In the case of selective boundary driven growth, we observe similar dynamics but with the ability of rejecting neutrality if differential selection of the growing subclone is captured within the punch/needle sample. We note that under selective boundary driven growth, the subclone often remains imprisoned, leading to neutral-like dynamics. Similar dynamics to Fig 2B are observed when positive selection is modelled as the probability of growing in the absence of space (increased pushing probability parameter *a*) rather than the increased birth rate. This leads to dynamics dominated by the homogeneous growth of the subclone rather than boundary growth of the background clone (S7 Fig). Moreover, removal of the majority of cells (99%) by treatment leads to enhancement of outgrowth of selected clones due to competitive release (S8 and S9 Figs).

**Fig 3:**
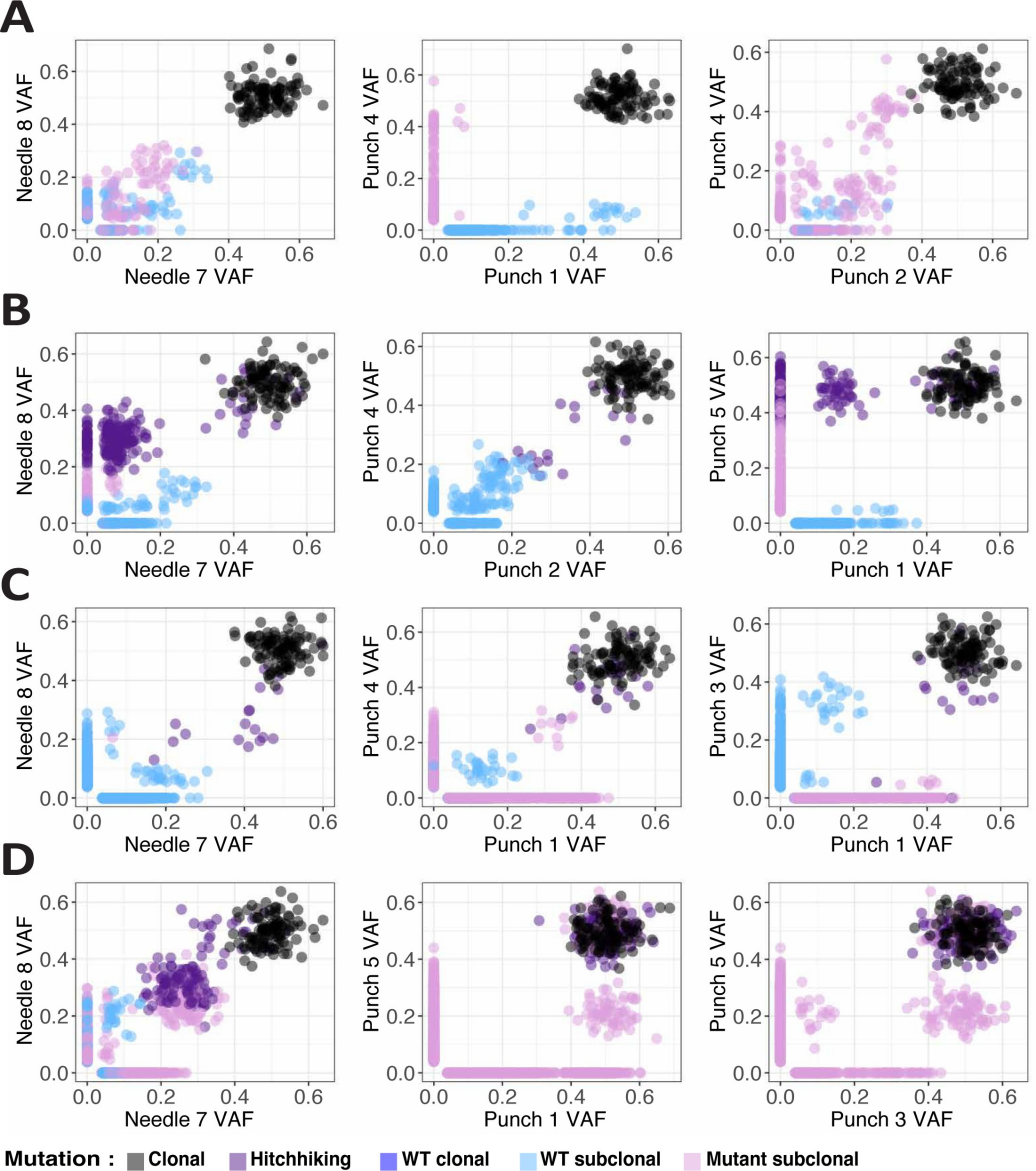
Sample vs sample scatterplots of mutations. For each of the representative cases: **(A)** neutral homogeneous, **(B)** selective homogeneous, **(C)** neutral boundary driven, **(D)** selective boundary driven, we report the scatterplots of somatic mutations in selected samples. Clearly, the presence of passenger subclonal mutations in the neutral tail of growing clones that spread in space as the tumour grows produces scattered variants (e.g. A). Even more striking is the formation of subclonal clusters of mutations particularly in the presence of boundary driven growth (e.g. C, D) where some lineages are over-represented not because of differential selection, but because of sampling bias and spatial drift.

We then looked at the pairwise VAF distributions between samples. The amount of subclonal mutations scattered through the frequency spectrum (Fig 3) and the number of subclonal clusters due to sampling bias and spatial drift was significant (e.g. Fig 3D). As per ground truth, only the dark purple mutations should show a subclonal clustering pattern (e.g. Fig 3B, punch 1). We found that scattered variants were mostly due to the effect of neutral lineages spreading in space, and then subsampled in different ways in each tumour region. In the case of boundary driven growth, sampling bias produces evident clusters that do not correspond to differently selected subclones in the tumour. This makes the reconstruction of the true clonal phylogeny and its evolutionary interpretation problematic.

### Spatial effects on single-cell sequencing

Most of the confounding factors we have described so far result from the limitations of bulk sequencing, where the genomes of many cells are convolved within samples. Single-cell sequencing does not suffer from this particular limitation and promises high-resolution cancer evolutionary analysis devoid of the drawbacks of bulk sequencing [43].

**Fig 4:**
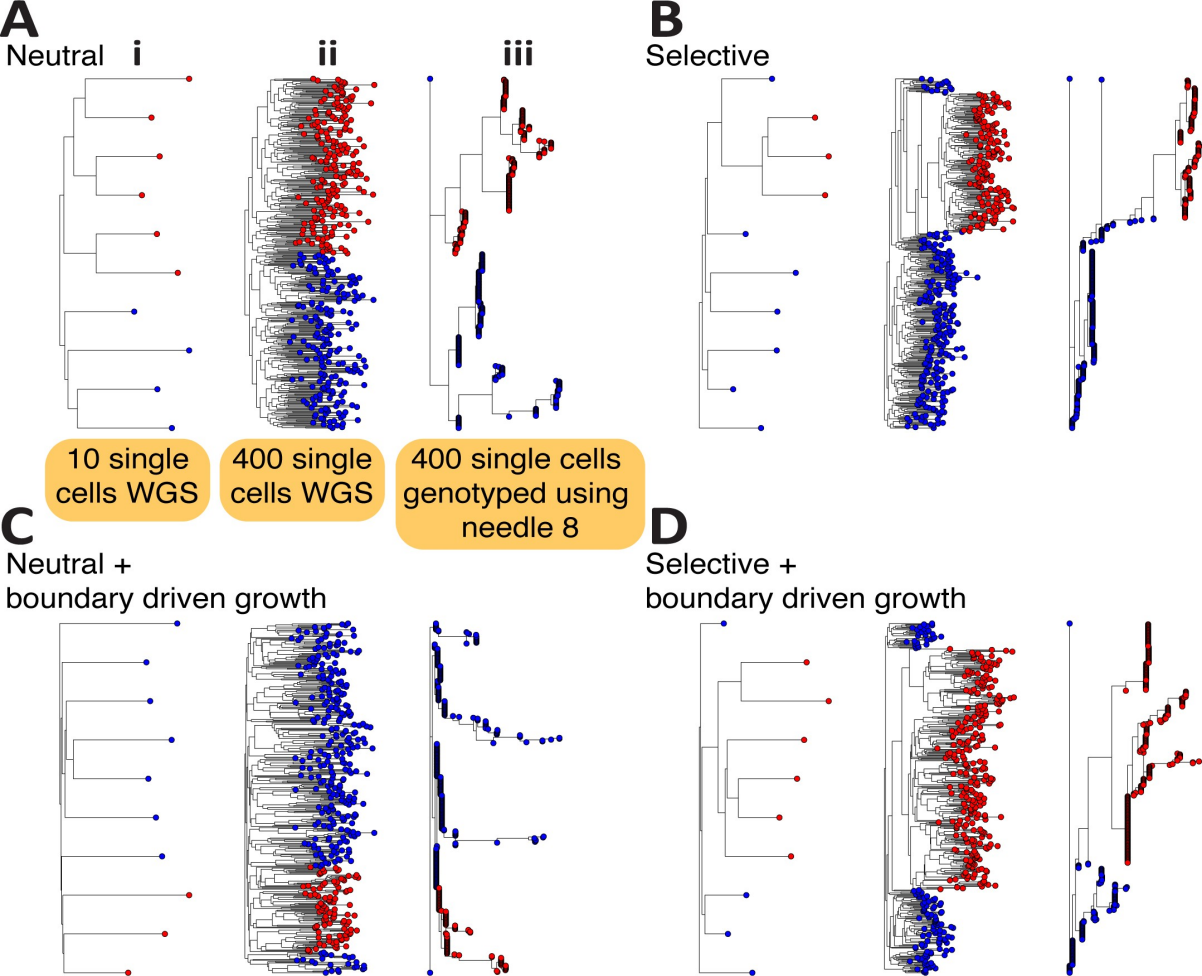
Single-cell sequencing data from spatial tumour simulations. **(A)** From each representative scenario we sampled 10 single-cells at random (i) as well as 400 single-cells at random (ii) and performed synthetic whole-genome sequencing. In both homogeneous **(A)** and boundary driven growth **(C)**, single-cell sequencing significantly reduces the sampling bias that we found in bulk samples and the only overrepresented lineages were due to selection **(B, D)**. However, due to the currently high error rate of single-cell sequencing, several studies rely on single-cell genotyping using mutations found in bulks. We simulated this by genotyping on 400 single-cells the mutations found at VAF>5% in needle biopsy 8 of each tumour (iii). The resulting trees are hard to interpret in terms of the clonal phylogeny due to the bias in the selection of variants to be genotyped.

To examine the effect of single cell sequencing, we simulated whole-genome sequencing of 10 single cells taken at random from the tumour and reconstructed their phylogenetic relationship (Fig 4A-i). For the neutral cases (Fig 4A and 4C), the patterns are consistent with a typical 'balanced' neutral tree, wherein all lineages contribute roughly equally to the final cell populations. In a balanced tree, the average distance between the trunk and each leaf of the tree is similar in each lineage. In cases with selection (Fig 4B-i and 4D-i), the selected subclonal lineages are over-represented on the tree (as reflected in VAF distributions), as the red lineage is introduced at time t = 8 and would have been much smaller if it was not selected for. Here the average distance between trunk and any leaf is different in the background vs the new clone. The pattern is even clearer if we sample 400 single cells and performed WGS (Fig 4B-ii and 4D-ii). We note that if we use randomly sampled single cell sequencing and plot the site frequency spectrum (frequency distribution of mutations within the population of sampled cells) we recapitulate the VAF distribution, including subclonal clusters and 1/f^2^ tails (S10 Fig). This is because the site-frequency spectra derived from single-cell sequencing data corresponds to a VAF distribution.

However, as whole-genome mutational profiling of single cells is still difficult due to allele dropout [44], often single-cell genotyping has to be performed instead [45]. In this approach, a bulk sample is sequenced and all mutations in that bulk sample are then tested in single cells for presence/absence of the mutation. Integrating bulk sequencing with single-cell information is extremely powerful [46], but requires careful interpretation of the results. In Fig 4A-iii we show that this approach, although informative, can lead to very distorted phylogenetic trees where branch lengths are heavily biased by the initial choice of mutations to be assayed, and consequently the signature of selection vs neutrality is not readily identifiable from these data alone.

**Fig 5:**
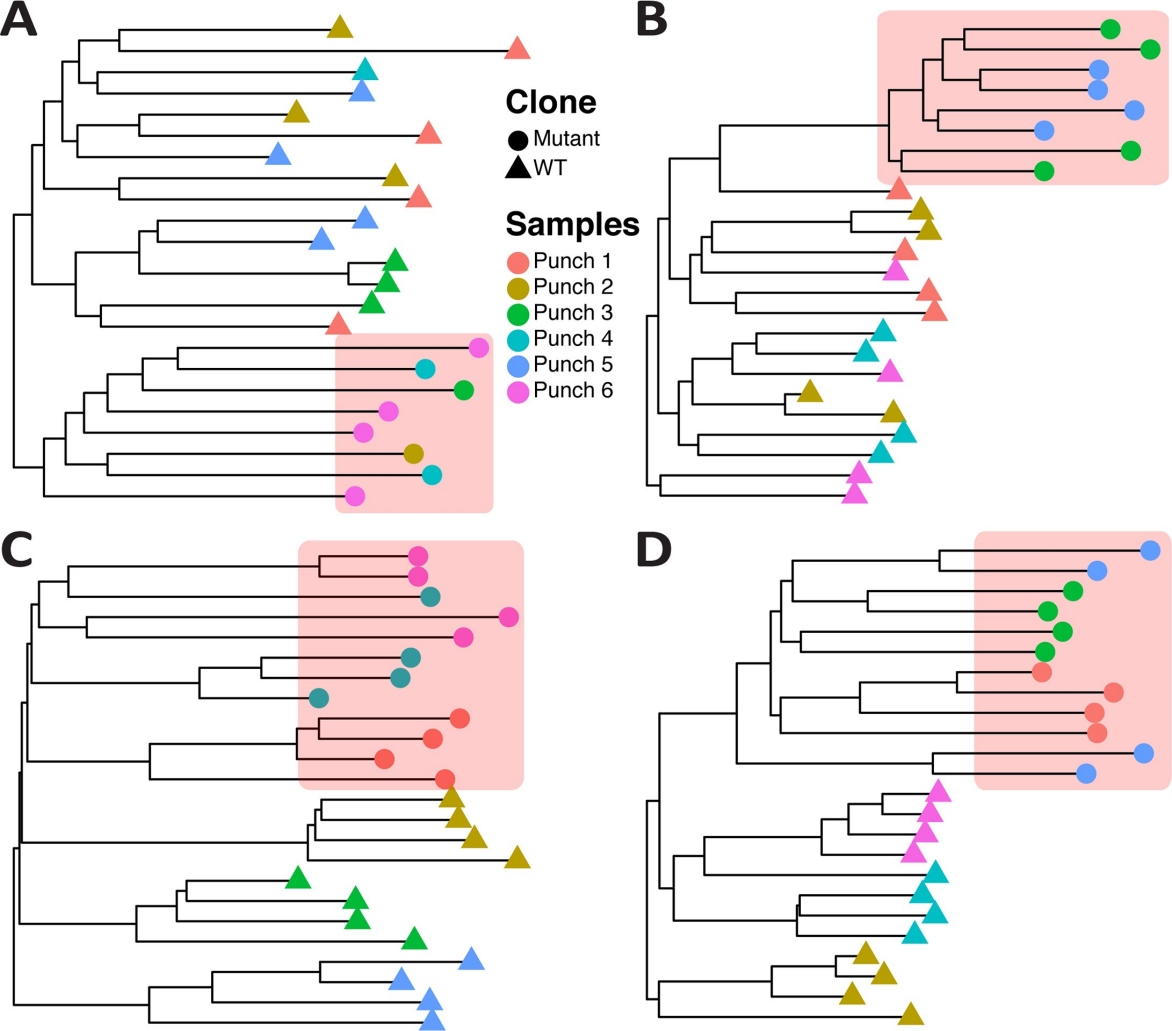
Biases of single-cell sequencing when cells are taken from spatially separated bulk samples. Whereas taking N random cells from a tumour highly reduces sampling bias, this is often not how single-cell from neoplasms are sampled. Often first small chunks of the tumour are dissected and then single-cells are isolated from those. **(A)** neutral homogeneous, **(B)** selective homogeneous, **(C)** neutral boundary driven, **(D)** selective boundary driven. For each of our representative examples, we simulated this type of sampling and show how this impacts severely on the phylogenetic tree and patterns of clonal intermixing. In particular, single-cell sampling from bulks alters the detected phylogenetic relationship of the cells because, since groups of cells come from spatially segregated regions, those appear more closely related than expected by chance. This is an important source of sampling bias that needs to be considered when analysing single-cell phylogenies. Cells coming from the ‘red’ mutant subclone are highlighted in the red shaded box.

Moreover, significant sampling bias is still apparent for single-cell sequencing when individual cells are not sampled uniformly at random from the whole tumour, but instead isolated in ‘clumps’ from different bulk samples. In Fig 5 we have simulated the collection of 4 single cells from each of the 6 punch biopsies in Fig 2 (these are the same simulations used to generate Fig 4). The trees are quite different from those sampled in Fig 4 and moreover, it is interesting to see how the underlying patterns of growth are reflected in the mixing of cells from different bulks. For instance, homogeneous growth leads to very high intermixing of cells in different bulks, whereas boundary driven growth tends to spatially segregate bulks. We have quantified the level of intermixing for different modes of growth in all our simulation cohort, highlighting this pattern (S11 Fig). We have observed these patterns in real data from carcinomas vs adenomas, where carcinomas were characterised by clonal intermixing, but adenomas were not [47]. Similar patterns of intermixing have also been found more recently using single-cell seeded organoid sequencing [48].

### Resolving spatial effects with inference

The spatial effects of drift and sampling bias one can observe are remarkable and represent a major challenge for the correct subclonal reconstruction of tumours growing in three-dimensional space. Due to the inherent complexity, analytical solutions to this problem that take space into the account remain challenging, although some attempts to tackle this difficult question are being undertaken [49]. Understanding the complex impact of spatially growing cell populations on the actual genomic data requires an approach based on computational simulations.

**Fig 6:**
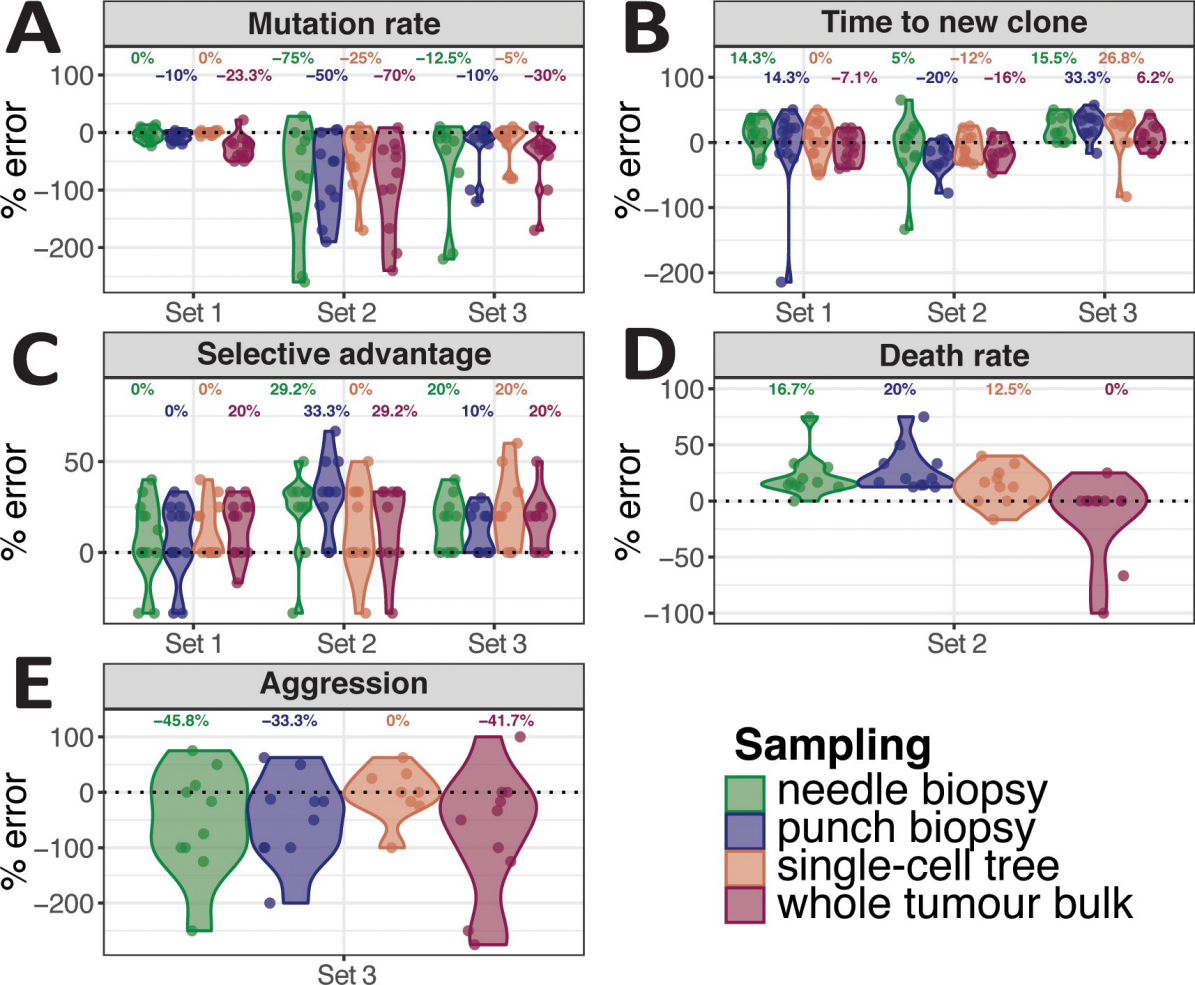
Statistical inference framework to recover evolutionary parameters. We combined our model with a statistical inference framework (Approximate Bayesian Computation–Sequential Monte Carlo) in order to infer the evolutionary parameters of selection and growth from the data. We tested this framework on 34 synthetic (target) tumours for which we generated genomic data. Our of these 34 target cases, 13 were characterised by homogeneous growth with no cell death **(A, Set 1)**, 11 were homogeneous but with cell death **(B, Set 2)**, and 10 where characterised by boundary driven growth **(C, Set 3)**, see all parameters in S1 Table. We tested the ability to recovery parameters of 4 different sampling schemes: punch samples, needle biopsies, single cell phylogenetic trees and whole-tumour sampling (see Materials and Methods for details). We report the percentage error of the inference (true parameter value–inferred value based on the mode of the posterior probability) for each parameter and scenario. See prior parameter ranges in S2 Table. **(D)** For the homogeneous stochastic cell death scenario (Set 2), we also report the error in recovering the death rate parameter d. **(E)** For the boundary driven growth scenario we report the error in recovering boundary driven growth parameter a (Set 3).

Here we devise a statistical inference framework, similar in spirit to what we previously proposed for well mixed populations [25], that aims at recovering the evolutionary parameters of each individual tumour from the type of data we have discussed so far (see Materials and Methods for details). We constructed a test-set of 34 synthetic tumours simulated with different parameters (see S1 Table) and assessed the error in recovering the parameters used to generate these tumours after statistical inference with an Approximate Bayesian Computation–Sampling Monte Carlo (ABC-SMC) approach [25,50–52]. The details of the inference algorithm are detailed in Material and Methods. We used approximately one million simulation instances to perform parameter inference using priors in S2 Table. We were particularly interested in comparing the accuracy provided by the different spatial sampling methods in recovery evolutionary dynamics. We studied three different sets of tumours. In the first set, we investigated parameter recovery in tumours with homogeneous (exponential) growth, with and without selection but with no cell death. In the second set, we added stochastic cell death as an additional factor. In the third set, we studied cases of boundary driven growth where we also examined our ability to recover the extent of the boundary driven parameter *a*. In all three sets, we studied the differences in the ability to recover parameter if we used a single bulk sample of the whole tumour multi-region punch biopsies, multi-region needle biopsies or single-cell sequencing. Following the inference of the parameters, we calculate the percentage error for each parameter as a difference between the true parameter value and inferred parameter value (mode of a parameter posterior distribution) scaled by the true parameter value. Then we plot the distributions of the percentage errors for each parameter per growth model and sampling strategy in Fig 6.

Not surprisingly, the scenario with exponential homogeneous growth without cell death was the one where the evolutionary parameters were the easiest to recover because spatial constrains were limited and the number of unknown parameters lowest (Fig 6A–6C, “Set 1”). In particular, the percentage-error in recovering the mutation rate *u* was particularly low, especially using single-cell sequencing (Fig 6A, “Set 1”). The mean percent error of the parameters *t* (Gillespie time when a new mutant is introduced) and *s* (selective coefficient of the new mutant), in the case of homogeneous growth were also within 20% and overall agrees with our previous observations in well-mixed populations [25]. The presence of stochastic cell death, even within a homogeneously growing tumour, introduced significant spatial and sampling biases (spatial drift) that led to a higher error in the recovery of the parameters (Fig 6A–6C, “Set 2”). Furthermore, some of the evolutionary parameters became unidentifiable (mutation and death rate). In this scenario, the best sampling strategies to recovery the death parameter *d* were single-cell sequencing or whole-tumour sequencing, reflecting the need to collect large population of cells for the correct estimation of this parameter (Fig 6D). Boundary driven growth also introduced significant biases that led to higher percent-error values in the recovered parameters (Fig 6A–6C, “Set 3”). Here, single-cell sequencing was best in recovering the boundary driven growth parameter *a* (Fig 6E). See S12 Fig for summary statistics from the simulations in Fig 6. The full posterior distributions of each parameter in each context is reported in S13 Fig. Parameter dependency in the inference of *t* and *s* combinations is reported in S14 Fig. We performed the same inference approach but with 3-dimensionally growing tumours using a test set of a single simulated ‘target’ tumour and inferred the parameters using approximately 10 million simulated cancers and found similar results (S15 Fig). We do recognise that for complex scenarios that are heavily affected by spatial constrains, such as boundary driven growth, inferred parameter values still suffer from high uncertainty in our ABC framework. This suggests the need for further model development and generation of higher resolution data for high confidence estimation of evolutionary parameters in cancer.

## Discussion

It is now widely accepted that tumour growth is governed by evolutionary principles. Thus, recovering the evolutionary histories of tumours is essential to the understanding patient-specific tumour growth and treatment response. However, these analyses are inevitably based on limited information due to sampling biases, noise of known and unknown nature, lack of time resolved data amongst many others. Despite these limitations, many approaches based on single sampling, multi-region bulk profiling, or single cell sequencing have been developed. Information from such data is often derived using purely statistical bioinformatics methods such as clustering analyses, without consideration of the confounding underlying influence of the cellular mechanics of tumour growth. Here we explicitly investigated spatial effects on the evolutionary interpretation of typical multi-region sequencing data of tumours. We found that the effects of sampling bias and spatial distributions of spatially intermixed cell populations critically depend on the mode of tumour growth as well as the details of the underlying sampling and data generation procedure. Most surprisingly, we could observe clusters of over-represented alleles in the VAF distribution of some tumour samples that were indistinguishable from positively selected subclonal populations, despite emerging solely due to the spatial distribution of cells. Such clusters vary depending on how one samples a tumour, and would therefore cause a major challenge for the evolutionary interpretation of cancer genomic data based on subclonal reconstruction.

We furthermore presented a Bayesian inference framework to recover evolutionary parameters from our spatial distributions. Evolutionary parameters such as strength of selection or mutation rates may be important surrogate measurements of evolvability, and hence linked to progression and treatment resistance, as it has been demonstrated for the rates of chromosomal instability [53,54]. Again, we observe that our ability to precisely recover certain evolutionary parameters depend on the scenarios of tumour growth and spatial sampling strategies. However, we do believe that although complex, the situation is far from hopeless. More involved statistical frameworks based on first principles of tumour growth can help resolving some of the evolutionary parameters on an individualised patient basis. Importantly, careful spatial sampling and single-cell sequencing can mitigate some of the confounding issues. We do acknowledge that our model has some important limitations, such as the infinite allele assumption (which could be violated by copy number loss [44]). We also recognise that we tested our inference framework only using our own generative model, and that despite the generative model matching the assumptions intrinsic to the inference the posterior parameter estimates still suffered from high uncertainty in some cases, reflecting the complexity of the problem. Also, for computational feasibility we mostly focus on 2D spatial analyses and of a relatively limited number of cells with respect to the billions of cells present in a human tumour. We also acknowledge that we do not offer a closed mathematical formulation for the distribution of alleles under spatial effects, which would be very useful but remains a very difficult problem that can only be tackled partially (e.g. [37]). Additionally, more realistic models of tumour growth dynamics that account for force fields between cells [55] have been developed that could improve on the study of spatial patterns of growth [23,56]. For computational feasibility, especially in regards to the necessity of performing statistical inference on the data and generate thousands of simulations, we restricted our analysis to the stochastic cellular automaton model we propose here. Nevertheless, our approach highlights the importance of spatial modelling of real data and the impact of confounding factor in our estimate and understanding of tumour evolution.

Importantly, future versions of the model could help guiding optimal sample collection that would minimise the spatial biases in the data. Due to the current technical limitations of these types of approaches, we are still far from direct application in the clinic. Additional effort should also be directed towards the use of measurements from other clinical data, such as imaging, where estimations of necrosis for example, can help parameterise computational models. However, we argue it remains extremely important to understand the confounding factors and spatial biases we expect to find in samples from which often we need to base clinical decisions on. Mathematical modelling of cancer evolution is a growing field with a fast expanding repertoire of models and approaches [11,57], however attention to clinical and biological relevance of modelling approaches is necessary to ensure these efforts are not dead ends.

## Materials and methods

### Details of the model

We developed a computational stochastic model of spatial tumour growth that allows simulating different strategies of multi-region tissue sampling followed by synthetic generation of high-throughput sequencing data. We consider tumour cells as asexually reproducing individuals that die and divide with certain pre-defined probabilities. If *b* is the birth rate for each cell and *d* the death rate, then the growth of the population over time *t* is:

$$N(t)=e^{(b-d)t}$$ [1]

where *N(t)* is a population size at time *t*, and *b-d* is the net growth rate. At first, we assume that birth and death rates are constant over time, whereas the overall growth rate can vary over time due to the randomness of each birth or death event, as well as due to spatial constrains that can limit or promote cell division over time. We model spatial constraints with the boundary proliferation parameter *a*, which models the distance from the border of the tumour within which cells are allowed to proliferate even in the absence of space (by pushing neighbouring cells outwards). When *a*~1 all cells can proliferate (homogeneous growth), and their growth is equivalent to an exponential expansion. When *a*~0, cells can only proliferate if they have an empty space in their neighbourhood, resulting in only a small layer of cells at the tumour border being able to divide. In this case the growth curve can significantly deviate from Eq [1].

In addition to cell division, we also model mutation and selection, where the latter can change birth and/or death rates. We model somatic mutations acquired by each cell after division as a Poisson random variable – Pois(*u*), where *u* is the mean mutation rate. Thus, after each cell division, a random set of new unique mutations occur in each cell of the two cells resulting from the division. The majority of these mutations are passenger mutations and hence do not affect a cell’s phenotype. However, they enable us to trace cell lineages uniquely in the final tumour. In addition, we also allow for driver mutation ‘events’ that can lead to positive selection of a subpopulation of cancer cells: a driver event conveys a fitness advantage to that particular cell and its offspring, thus allowing the lineage to increase in frequency. Since we ask what is the distribution of mutations across space, rather than the expected waiting time of driver events as previously analysed [58], we introduce a driver mutation at a fixed time in our simulations, also to make simulations comparable and computationally efficient.

To simulate tumour growth in space with these four stochastic events–birth, death, mutation and selection–we have used a modification of the Gillespie algorithm [26].

Specifically, the simulation framework works as follows:

- **Initialization:** start with a 2D/3D grid with Von Neumann neighbourhood. Place the first tumour cell in the centre of the grid. Set time *t = 0*.

Until a cell reaches a predefined grid boundary, repeat the following steps

1. Compute the reaction propensities according to the Gillespie algorithm. Each reaction event of birth (or death) has a functional form *f*(*x*) = *kx*; where *x* is the number of cells of type “x” (wild-type or mutant), and *k* is either the birth or death rate. The time of each event is obtained by sampling an exponential random variable with mean given by its propensity. The next event chosen is the one completing first (i.e., with the smallest clock value, as in the so-called next reaction method [26]). Given the event, we increment the time by its clock. Note that these time steps do not correspond to population doubling times i.e. generations; doubling times can be retrieved scaling time by a factor log(2).
2. If the next event is a cell division, we use a heuristic method to place the 2 daughter cells on the grid. We first replace the parent cell with the first daughter, and search for a suitable position to place the second daughter cell. We use a Von Neumann neighbourhood and check if any of the 8 (in 2D grid) neighbouring spots of the parent cell is empty; if one or more are, we locate the second cell in one of those spots at random. Otherwise, with a probability determined by a parameter *a*, we push all cells along a randomly chosen direction until we hit the grid boundary, and place the second daughter at the nearest emptied spot. With the parameter *a* we can model boundary driven growth, as it represents the fraction of the radius of the growing tumour where cells are allowed to proliferate; that is, *a* = 0.2 creates a tumour periphery of width equal to 20% of the whole tumour width in which cells are allowed to proliferate even without empty space by pushing neighbouring cells outwards (when *a* = 1, periphery width is 100%, every cell can always push and divide, and the tumour grows exponentially). When a cell divides, we generate passenger mutations by drawing a number from Pois(*u*). These mutations will be assigned to both daughter cells.
3. If the next event is cell death, we simply free the position allocated to the cell.
4. At the end of this step, we check if the clock is greater than the time of the next scheduled driver event *t*_*driver*_; if it is, we convert a single wild type (WT) cell into a new mutant and increase its birth rate, or decrease its death rate. This will result in mutant cells having a proliferative advantage. To quantify the effect, we define the fitness *s* as: 1 + *s* = (*birth_mutant* – *death_mutant*)/(*birth_wt* – *death_wt*).

**Details of the data generation and error modelling.** At the end of the simulation, we can collect bulk or single-cells and simulate sequencing data generation. Bulk Samples are spatially separated tumour chunks ‘cut out’ from the tumour. We model two different shapes:

1. Squares, which are referred to in the paper as ‘punch biopsies’
2. Long thin rectangles that resemble a ‘needle biopsy’

A bulk sample is a set of adjacent cells from the final tumour population. Each cell has its unique ID, a position on a grid and its list of somatic mutations. From the sampled cells (in a bulk) joined list of mutations we can construct the Variant Allele Frequency (VAF) distribution as in a real sequencing experiment.

To construct a VAF distribution from a simulated bulk tumour sample, we mimic realistic next generation sequencing steps, specifically sequencing coverage and limits of detectability of low frequency mutations. We proceed as follows:

1. We generate (dispersed) coverage values for the input mutations by sampling a coverage from a Poisson distribution *D*~*Poisson*(*λ* = *Z*) with mean *λ* equal to a desired sequencing depth.
2. Once we have sampled a depth value *k* for a mutation, we sample its frequency (number of reads with the variant allele) with a Binomial trial. We use *f*~Binomial(*n*,*k*) where *n* is the proportion of cells carrying this mutation in the sample.

This procedure guarantees that the generated read counts reflect the proportions of mutations in the simulated tumour. To model limits of detection of a mutation, after resampling a mutation, we discard it if the corresponding number of reads containing the variant allele is less than 5 (using the fixed coverage 100, which accounts for a ~0.05 minimum VAF).

We also performed single cell sequencing taking either random single cells across the whole tumour population, or from spatially structured biopsies (mimicking bulk tissue collection followed by single-cell isolation). We used the obtained single cells to construct maximum parsimony phylogenetic trees. In addition to single cell sequencing, we also model genotyping cells with a given list of mutations, corresponding to targeted sequencing of mutations found using e.g. exome or whole-genome sequencing. To implement this, we take one of the bulk samples as reference genotype and check for the presence of each individual mutation in a random set of 200 cells. Similarly, we use the obtained genotyped single cells to infer phylogenetic trees and check how much the genotyped trees differ from the single cell trees.

### Details of the ABC framework

Due to the complexity captured by our spatial model of tumour growth, we do not have explicit formulas for the stationary probabilities of the stochastic process, and hence cannot derive a likelihood function. Thus, we have to use likelihood-free methods to perform statistical inference on the parameters and compute the posterior distribution of the parameters ***θ***.

Here we use Approximate Bayesian Computation (ABC) [51,59] to infer the parameters of our model. ABC is based on the idea of scanning a large grid of plausible values for ***θ***, and simulating the model many times with such parameters. Outputs of the model are stored and compared using a predefined set of summary statistics that are initially evaluated on real data. We can then rank sets of parameters that lead to the generation of synthetic data that are close to the observed data. We can estimate a posterior distribution *p*(***θ***|***D***) for the model parameters ***θ*,** using the available data ***D*** and the prior for ***θ***. This method is computationally intensive, and requires running several hundred (ideally thousands or millions) simulations. In our case we have generated ~74 million simulations that we use to perform the inference step.

There are different approaches to implement ABC, the simplest is rejection-sampling. More advanced implementations such as ABC with Markov Chain Monte Carlo (MCMC) can result in significant increases in efficiency. In our paper we implemented a simple rejection-sampling algorithm first, and then added Monte Carlo simulation techniques to speed up convergence. The simple ABC rejection-sampling algorithm consists of the following steps:

1. Sample parameter vector ***θ*** from a prior distribution *p*(***θ***).
2. Run the model with the given parameter set and generate the synthetic dataset
3. Evaluate the distance between the simulated dataset and the target data
4. If the distance is less than a desired threshold, accept the parameters.
5. Return to step 1 and repeat until *N* parameter values are accepted.

In this study we use uniform priors for all parameters: *u*~Uniform(0, 100), *s*,*d*,*a*~Uniform(0, 1), *t*_*driver*_~Uniform(0, 15). One of the most important factors that affect the ABC outcome is the number of simulations that one can afford to run, and the summary statistics were chosen to evaluate the distance between a target and a simulated dataset. Summary statistics can be any quantitative measurement that captures the information from the multidimensional data without losing too much information. As for our distance metric, we use Euclidean and Wasserstein distances between summary statistics for different parameters as discussed below.

Wasserstein metric estimates the distance between probability distributions by treating each distribution as a unit amount of dirt piled up on a given metric space and calculates the minimum cost required to convert one pile into another. If *x* and *y* are two vectors we want to evaluate the distance of, first we calculate their empirical distribution functions $F(t)=\sum_{i=1}^{m}w_{i}^{\left(x\right)}l\{x_{i}\leq t\}$ and $G(t)=\sum_{i=1}^{n}w_{i}^{\left(y\right)}l\{y_{i}\leq t\}$ (for weights $w_{i}^{x}$ and $w_{i}^{y}$ we took 1/*m* and 1/*n* respectively), the Wasserstein distance is defined by evaluating the following:

$$W_{p}(F,G)==(\int_{0}^{1}{\left|F^{-1}(u)-G^{-1}(u)\right|}^{p}{)}^{1/p}$$

where we took *p = 1* for our analysis. We used the R package transport (https://CRAN.R-project.org/package=transport) to implement the distance calculation.

We used different summary statistics for each sampling scheme. For punch, needle biopsy and the whole tumour sampling–we used the VAF distribution to compute our summary statistics. For the whole tumour VAFs, our ABC procedure was similar to the one in ref [25]. For the bulk samples, since our model implements multi-region sampling, we first evaluate the multivariate VAF distribution (which is a joint probability distribution of all sampled bulk VAFs) and then calculated the Euclidean distance between the obtained empirical probability distribution vectors:

$$D_{Euclidean}(F_{sim\_data}({VAF}_{bulk1},\ldots ,{VAF}_{bulkN}),F_{target\_data}({VAF}_{bulk1},\ldots ,{VAF}_{bulkN}),)$$

With single cell samples, we constructed phylogenetic trees per tumour and used different tree-based summary statistics to evaluate the distance. Since the inferred phylogenetic tree branch length is proportional to the number of unique mutations belonging to a node, we decided to compare the vectors of all branch lengths (between a simulated and target tumour trees) by computing the Wasserstein distance. For the subclone introduction time *t*_*driver*_, death rate *d* and the boundary driven growth parameter *a*, we chose to compare the vectors of branching times for each node of the phylogenetic trees.

Due to computational costs, we are limited to run the ABC framework with a small tumour size (~100k cells) or simulate smaller datasets per inference, both of which can significantly affect the outcome. To therefore speed up our ABC framework we implemented a Sequential Monte Carlo (SMC) algorithm to increase the acceptance rate of the simple ABC rejection algorithm. Our ABC SMC algorithm uses sequential importance sampling by running several rounds of resampling around the accepted parameters (correlating the rounds), and gradually decreasing the acceptance threshold while converging to the posterior distribution. This approach significantly increases the acceptance rate of the simulated datasets [60].

Our implementation of the ABC SMC algorithm is as follows:

1. Initialise the indicator to rounds *r* and the acceptance threshold *ε*
2. ***If*** *r* = 1
  2.1 Run the simple ABC rejection algorithm (described above).
  2.2 Order the simulated parameters set according to their corresponding distance values.
  2.3 Keep the top Q per cent of the parameters.
3. ***Else***
  3.1 Sample next particle *θ* = (*u*,*t*,*s*,*d*,*a*) from the accepted set of parameters from round *r* − 1 with weights *W*_*r*−1_.
  3.2 Perturb each sampled parameter *p*_*i*_ using uniform perturbation kernel *K* = *Unif*(*p*_*i*_ − *σ*,*p*_*i*_ + *σ*), where $\sigma =\frac{1}{2}(\max\left(p_{i}^{r-1}\right)-min(p_{i}^{r+1}))$.
  3.3 ***If*** *π*(*θ*) > 0, keep *θ*
    ***Else*** go to step 3.2.
  3.4 Simulate data from the model using the sampled particle *θ*.
  3.5 Calculate distance D between the target and the simulated data.
  3.6 ***If*** *D* < *ε*, keep *θ*
    ***Else*** go to step 3.1.
4. Calculate the weights for all accepted particles 1 ≤ *j* ≤ *N*:
  4.1 ***If*** *r* = 1, set *W*_(*j*,*r*)_ = 1
  4.2 **Else**
    $$W_{\left(j,r\right)}=\frac{\pi (\theta_{\left(j,r\right)})}{\sum_{l=1}^{N}W_{\left(l,r-1\right)}K(\theta_{\left(l,r\right)}|\theta_{\left(l,r-1\right)})}$$
5. Update the threshold *ε* to the top Q-th percentile of the accepted particles.
6. Repeat until *ε* is less than a desired convergence threshold.

Our ABC-SMC framework tries to recover all the parameters (referred to as a particle in the algorithm above) at the same time. We notice that once one of the parameters converges, the acceptance rate decreases significantly. We then decided to fix the converged parameter at the inferred value (mode of its posterior) and rerun the inference varying the rest of the parameters until other parameters converge, and repeat the procedure. We found that this significantly improved the convergence speed. For the 2D inference in Fig 6 we started with N = 100 simulated particles, performed r = 10 rounds with quantile Q = 0.5, leading to ~200k simulations for each parameter and ~1M simulations in total. For the 3D inference in S15 Fig we started with N = 1000 simulated particles, performed r = 10 rounds with quantile Q = 0.5, leading to ~2M simulations for each parameter and ~10M simulations in total.

### Input data format

The package implements three sampling strategies for the inference:

1. Bulk samples (punch or needle biopsies)—ABCSMCwithBulkSamples()
2. Single cell sample phylogenetic trees—ABCSMCwithTreeSampleBL() and ABCSMCwithTreeSampleBT() (using Branch Lengths or Branching Times as summary statistics)
3. Whole tumour bulk sample—ABCSMCwithWholeTumour()

Depending on the strategy, a user would need to provide real or synthetic target data in the form of tumour bulk sample VAFs (list of R data.frames where each row should correspond to a unique mutation with the following columns: clone (Clone type label set to 0), alt (Number of reads with the variant), depth (Sequencing depth), id (Unique mutation ID)), an array of whole tumour sample VAFs or single cell sampling phylogenetic trees. Alternatively, a user can provide a set of parameters (please refer to the package documentation for the details of each input parameter format) to simulate a synthetic target tumour to then recover these input parameters.

The functions output sequence of files containing sets of inferred parameters corresponding to each SMC round (that can then be used to construct the posterior distributions for each parameter).

### Phylogenetic tree reconstruction

For Fig 4 and parameter inference framework with single cell sequenced trees we used maximum parsimony phylogenetic algorithm implemented in paup [61]. For the genotyped phylogenetic trees in Fig 4, we manually constructed input genotype files for paup by recording presence/absence of a given mutation from the sampled 200 cells with respect to the reference mutations list (in our case mutations list taken from a bulk sample).

### Neutrality test

To test for the presence of selection and the mutation rate inference, we fit 1/f_2_ distribution to the empirical cumulative distributions of sampled VAFs using the R package developed in ref [25].

## Supporting information

S1 Fig Growth curves. Tumour cell population growth curves for each of the representative cases: **(A)** neutral homogeneous, **(B)** selective homogeneous, **(C)** neutral boundary driven, **(D)** selective boundary driven. Wild type (WT) and mutant growth curves are plotted separately in addition to the whole population growth curves. Without the spatial constraints of our model, the growth curves are exponential as expected. **(A, B)** With the boundary driven growth the growth becomes polynomial. We can also see for the tumours with selection **(B, D)** how the mutant subpopulation outcompetes wild type cell population.
(PNG)

S2 Fig Examples where selection is modelled by varying death rates instead of birth rates, and neutral growth under high cell death. Two examples where fitness advantage is modelled by decreasing cell death the mutant subpopulations and increasing for the wild type. **(A)** The death rate of the mutant subpopulation is 0.2 while for the WT is 0.8. **(B)** The death rate of the mutant subpopulation is 0.3 while for the WT is 0.9. **(C-F)** Examples of neutral growth with high cell death, which increases the level of genetic drift (especially noticeable in **(F)**) as well as the level of spatial intermixing due to stochasticity of cell replacement. Birth rate *b* was 1 in all simulations.
(PNG)

S3 Fig Mutational load comparison for different growth cases. **(A)** We sample each representative example tumours (T1 –neutral homogenous, T2 –selective homogenous, T3 –neutral boundary driven, T4 –selective boundary driven) from the tumour centre (bulk sample C1) towards the periphery following the concentric circles in four directions: W–west, E–east, N–north, S–south. The bulk indexes (2W, 3W, 4W) are proportional to the distance from the centre to the periphery. **(B)** We observe how the number of mutations per bulk sample increases proportionally to the distance from the tumour centre in the case of boundary driven growth. Also, the total number of mutations is much higher for the constrained boundary driven growth than for the homogenous tumour due to increased cell turnover in the former case.
(PNG)

S4 Fig Example of imprisonment. Example of selective boundary driven growth when the driver mutant subpopulation gets trapped within the wild type population despite being fitter than the WT clone.
(PNG)

S5 Fig The effect of stochasticity and sampling bias on the shapes of VAF distributions for the four representative scenarios. For each of the representative cases: **(A)** neutral homogeneous, **(B)** selective homogeneous, **(C)** neutral boundary driven, **(D)** selective boundary driven, we simulated 100 different runs of each case keeping the underlying parameters constant and varying only the random seed of the simulation. For each simulated tumour, we constructed needle and punch biopsy sample VAF distributions along with the whole tumour VAFs. Overall there is a less variation among the distributions for neutral **(A,C)** versus selective **(B,D)** cases. In addition, punch biopsy VAFs scatter more than needle biopsy samples in comparison to the whole tumour VAF distributions. **(E)** We separated the VAF distributions for the selective boundary driven between cases where the new clone escaped and grew to fixation, versus escaped by not yet fixed (signature of ongoing subclonal selection), versus imprisoned (leading to neutral dynamics)
(PNG)

S6 Fig Distribution of AUC based neutrality test p-values. **(A)** We simulate 100 different tumours for each 4 representative growth models and fit 1/f test to their corresponding whole tumour sample VAFs. Reported are the distributions of p-values obtained from each test using the AUC statistics. **(B)** For the cases of boundary-driven growth modes we compared tests of neutrality using the whole-tumour sample versus punch/needle biopsies.
(PNG)

S7 Fig Example of selection when mutant subpopulation has higher push power instead than higher birth rate. Example of a selective exponential growth when the mutant subpopulation has higher ‘push power’ than the wild type population.
(PNG)

S8 Fig Killing 99% of the cell population and re-growing tumours. For each of the representative cases: **(A)** neutral homogeneous, **(B)** selective homogeneous, **(C)** neutral boundary driven, **(D)** selective boundary driven, we simulated procedures of removing large cell population (here 99%) by the end of tumour growth and wait for it to regrow to its original size.
(PNG)

S9 Fig Growth curves through cell killing. Tumour cell population growth curves for each of the representative cases: **(A)** neutral homogeneous, **(B)** selective homogeneous, **(C)** neutral boundary driven, **(D)** selective boundary driven, where by the end of tumour growth we remove 99% of the cell population and wait for the tumour to regrow to its original size.
(PNG)

S10 Fig Allele frequency distributions derived from single cell sequencing. We construct the allele frequency distributions from sequencing the randomly sampled 400 single cells (same as in Fig 4) from the four representative tumour examples: T1 –neutral homogenous, T2 –selective homogenous, T3 –neutral boundary driven, T4 –selective boundary driven.
(PNG)

S11 Fig Distribution of Moran’s test effect size. We simulate 100 different tumours for each 4 representative growth models and test intermixing of subpopulations within each simulation lattice using Moran’s entropy-based test. Each individual test output significant p-values indicating to high spatial correlation between tumour cell types (mutant vs WT) and their location on tumour lattice. Although the test effect size (the observed values of the Moran’s test statistic) differ as we can see from their distributions per model scenario. The median values of each observed statistics are reported at the bottom of each violin plot.
(PNG)

S12 Fig Comparing the site frequency spectrum and phylogenetic tree balance index statistics for each representative scenario and sampling strategy. **(A)** Distributions of different summary statistics from single cell sampling (100x) phylogenetic trees for the four representative cases. The balance index-based statistics (sackin, colless with their different normalisation approaches–Yule, PDA) seem to have similar shapes among all four tumour cases, while tip and node Cophenetic distance-based statistics show different trends for neutral versus selective examples with not observable variation between homogenous and boundary driven tumours. Branch length-based statistics give similar results as cophenetic distances. Only one statistic, maximum node depth, tend to have longer flat tails for boundary driven tumours compared to homogenous tumour simulations. **(B)** For each of four tumour examples, we compare the total number of passenger mutations and final population sizes along with the time the simulations finish and the final frequency of the new sub-population (introduced after a driver event).
(PNG)

S13 Fig Posterior distributions of model parameters from each synthetic tumour. The violin plots of the posterior distributions for each model parameter per synthetic tumour inferred by our ABC-SMC framework. The three sets of tumours corresponding to the three tumour growth scenarios are plotted separately: exponential **(A)**, death **(B)** and boundary driven **(C)**. The number on the violin plots is the target value of each parameter.
(PNG)

S14 Fig The effect of stochasticity on the dependence of t and s parameter combinations on the VAF distribution. To explore the interdependence of the parameter pair *t* and *s*, for their different values we simulate tumour growth while fixing all the other parameters (2D grid size = 400, u = 10, d = 0, a = 1). We summarised the obtained tumours by calculating either the Euclidean norm of the obtained whole tumour VAFs **(C, D)** or the calculating Euclidean distance between the cumulative VAF distributions of the simulated and a chosen target tumour (in this case target tumour parameters are t = 7, s = 3) **(A, B).** To reduce the effect of stochasticity we fix the random seed in **(B)** and **(D)** and they indeed showed less scattered patterns of **(A)** and **(C)** plots respectively.
(PNG)

S15 Fig Posterior distributions for a 3D model. ABC SMC inference for a selective homogenous growth simulation in 3D space. Real ‘target’ values are reported as dashed lines. We run this ABC framework similarly to 2D simulations, where we recover each parameter at a time; first varying all parameters, once one is converged, fixing it at its inferred value and rerunning the simulation varying the parameters left to infer. Here we first recovered mutation rate, then time and selective advantage (together), and finally death rate and aggression (together as well). Similar to 2D models, our ABC framework with whole tumour sampling performs the best compared to other sampling strategies.
(PNG)

S1 Table Parameters of the set of synthetic tumours used to test the ABC inference framework. (CSV)

S2 Table Prior parameter ranges used for the synthetic ABC inference testing. (CSV)
