# Supplementary figures and images for: Spatially constrained tumour growth affects the patterns of clonal selection and neutral drift in cancer genomic data
Source: PLoS Comput Biol. 2019 Jul 29;15(7):e1007243. doi: 10.1371/journal.pcbi.1007243 (PMC6687187; doi:10.1371/journal.pcbi.1007243)

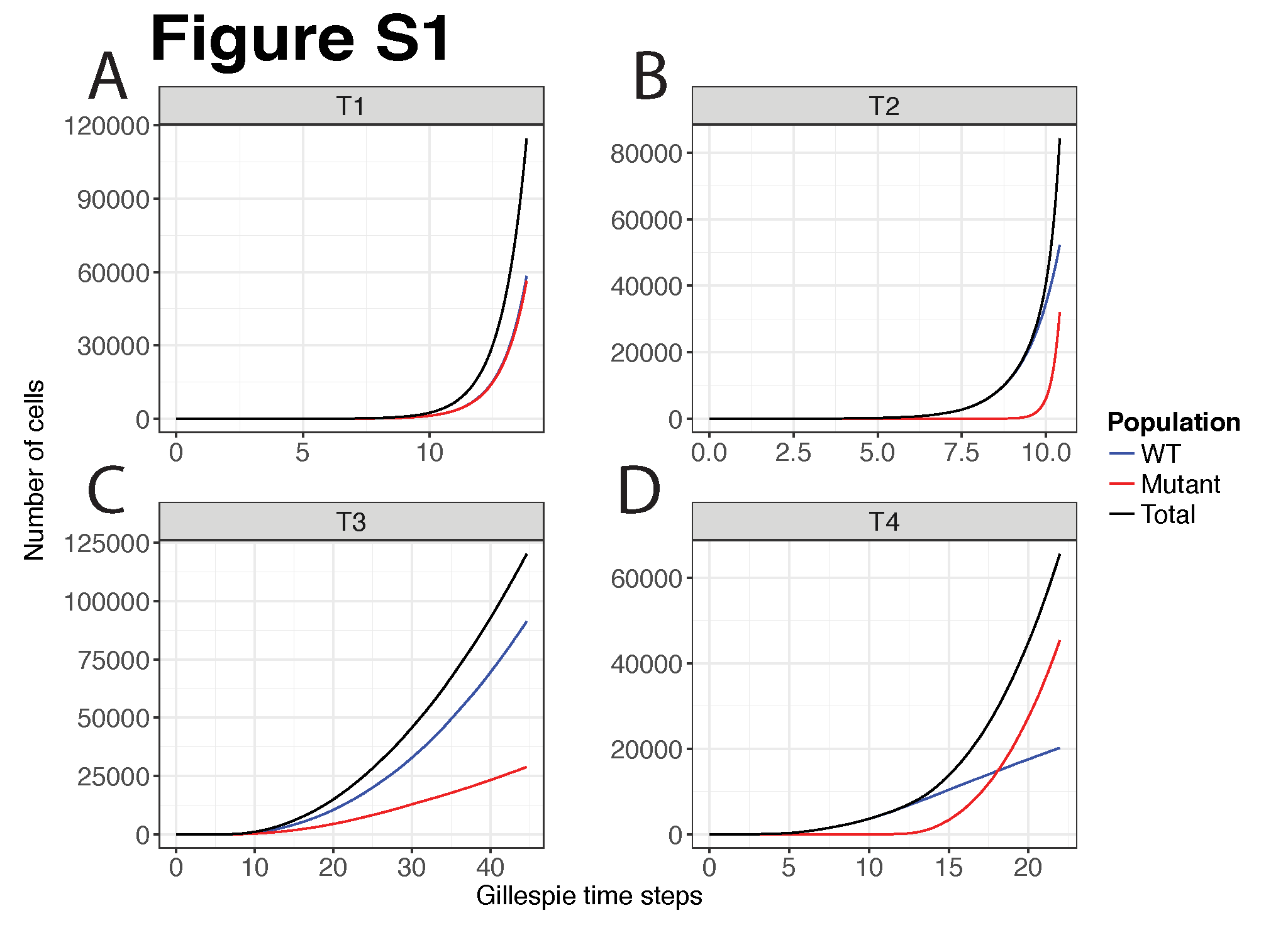

Supplement: S1 Fig — Tumour cell population growth curves for each of the representative cases: (A) neutral homogeneous, (B) selective homogeneous, (C) neutral boundary driven, (D) selective boundary driven. Wild type (WT) and mutant growth curves are plotted separately in addition to the whole population growth curves. Without the spatial constraints of our model, the growth curves are exponential as expected. (A, B) With the boundary driven growth the growth becomes polynomial. We can also see for the tumours with selection (B, D) how the mutant subpopulation outcompetes wild type cell population. (PNG) [file pcbi.1007243.s001.png]

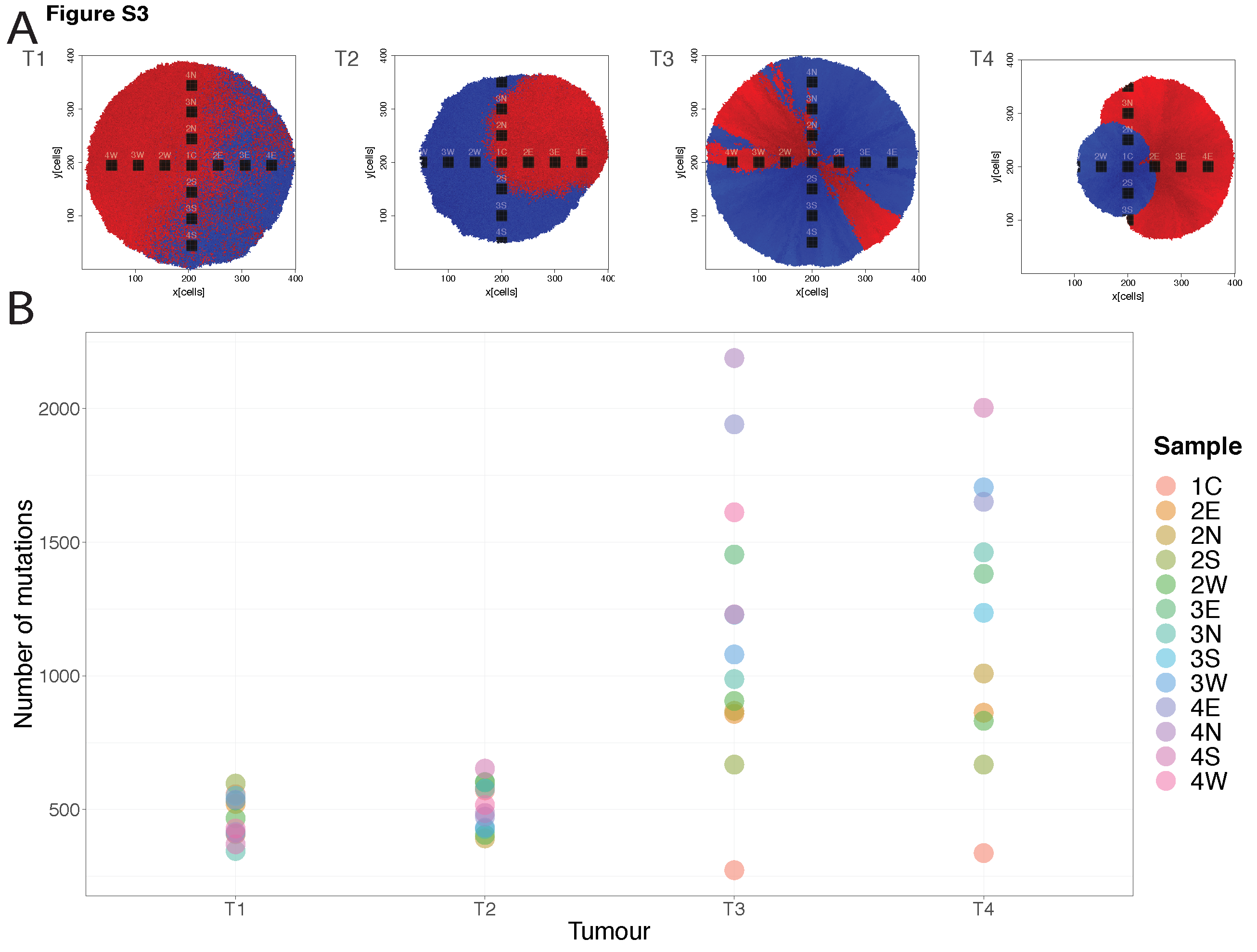

Supplement: S3 Fig — (A) We sample each representative example tumours (T1 –neutral homogenous, T2 –selective homogenous, T3 –neutral boundary driven, T4 –selective boundary driven) from the tumour centre (bulk sample C1) towards the periphery following the concentric circles in four directions: W–west, E–east, N–north, S–south. The bulk indexes (2W, 3W, 4W) are proportional to the distance from the centre to the periphery. (B) We observe how the number of mutations per bulk sample increases proportionally to the distance from the tumour centre in the case of boundary driven growth. Also, the total number of mutations is much higher for the constrained boundary driven growth than for the homogenous tumour due to increased cell turnover in the former case. (PNG) [file pcbi.1007243.s003.png]

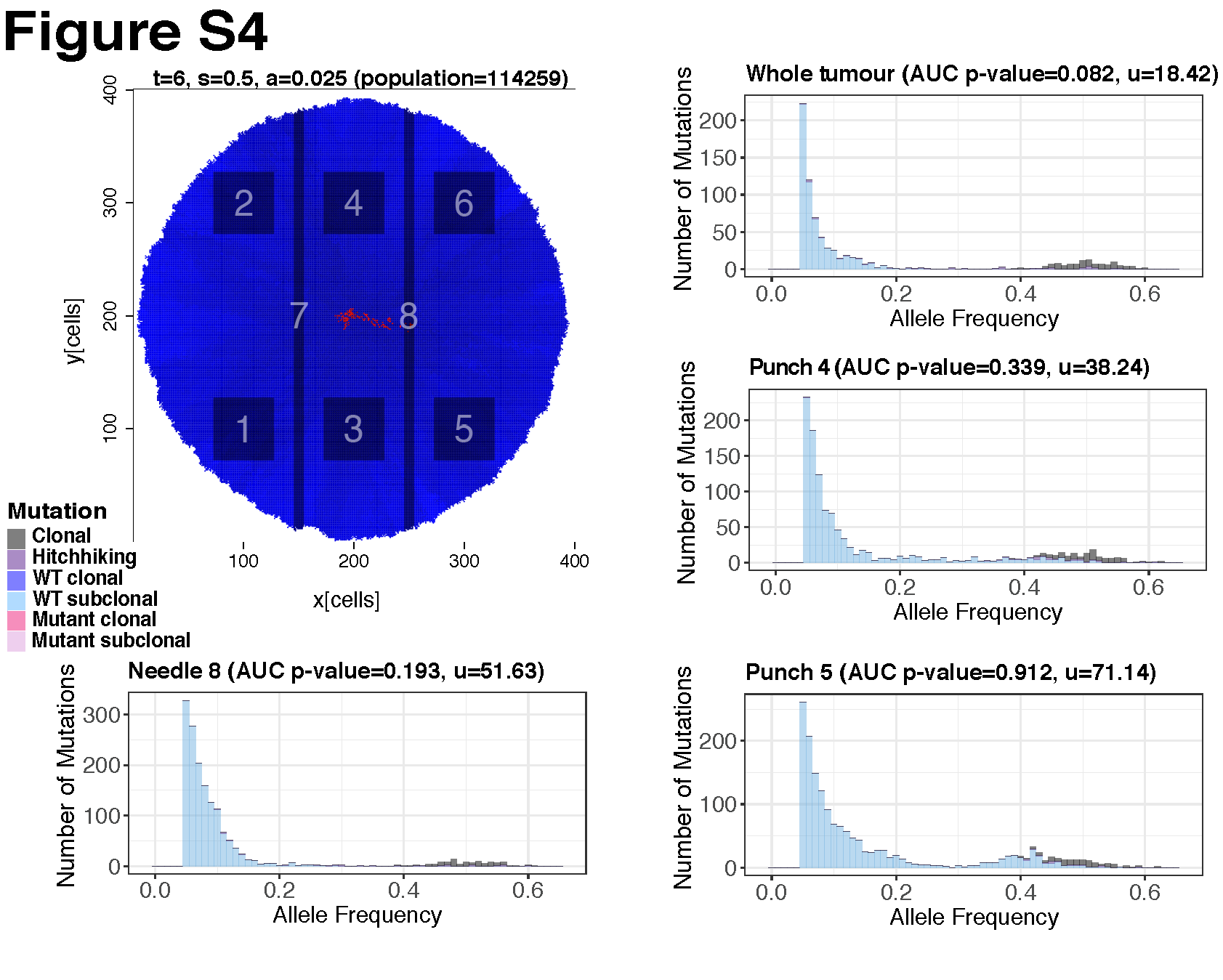

Supplement: S4 Fig — Example of selective boundary driven growth when the driver mutant subpopulation gets trapped within the wild type population despite being fitter than the WT clone. (PNG) [file pcbi.1007243.s004.png]

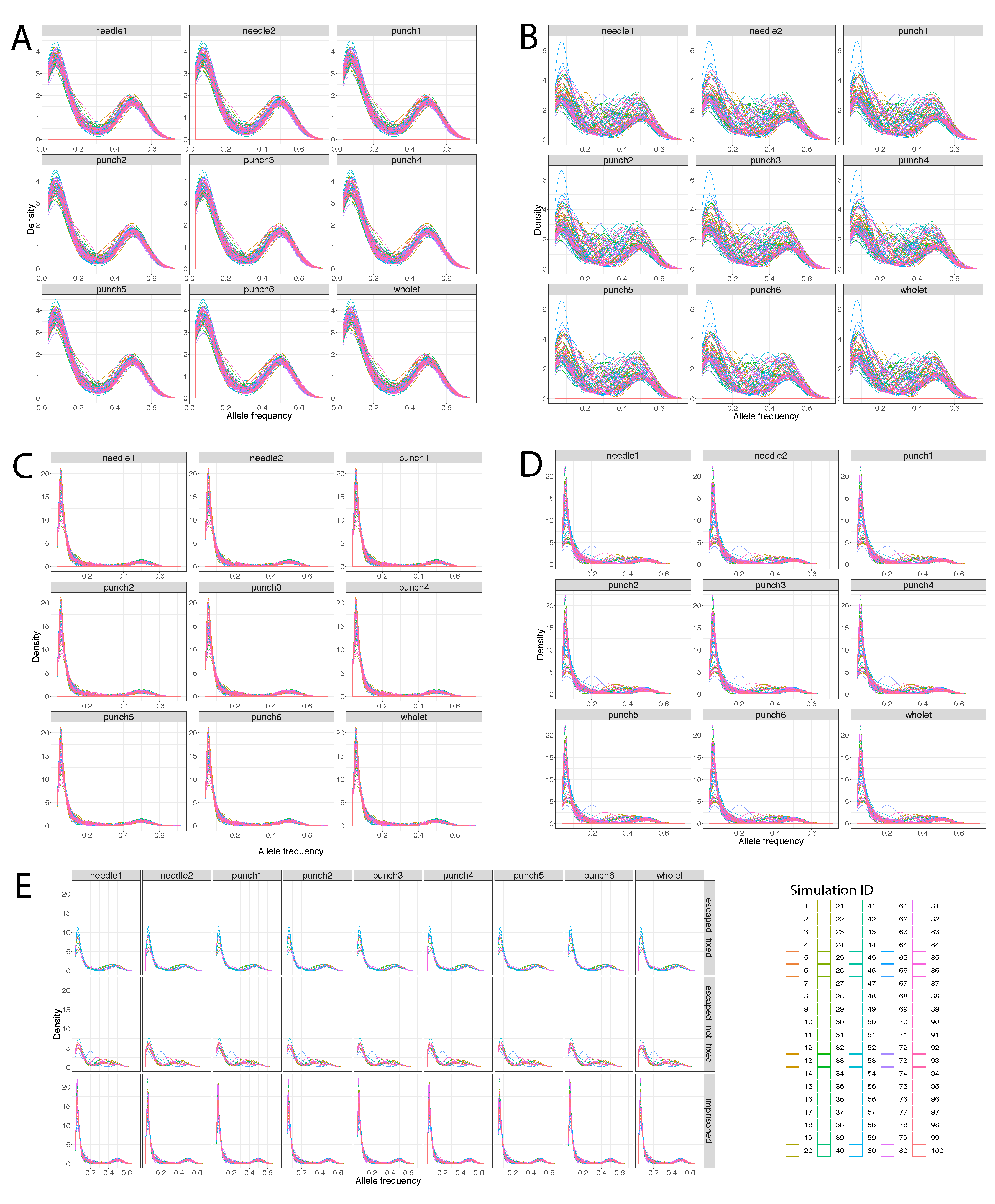

Supplement: S5 Fig — For each of the representative cases: (A) neutral homogeneous, (B) selective homogeneous, (C) neutral boundary driven, (D) selective boundary driven, we simulated 100 different runs of each case keeping the underlying parameters constant and varying only the random seed of the simulation. For each simulated tumour, we constructed needle and punch biopsy sample VAF distributions along with the whole tumour VAFs. Overall there is a less variation among the distributions for neutral (A,C) versus selective (B,D) cases. In addition, punch biopsy VAFs scatter more than needle biopsy samples in comparison to the whole tumour VAF distributions. (E) We separated the VAF distributions for the selective boundary driven between cases where the new clone escaped and grew to fixation, versus escaped by not yet fixed (signature of ongoing subclonal selection), versus imprisoned (leading to neutral dynamics) (PNG) [file pcbi.1007243.s005.png]

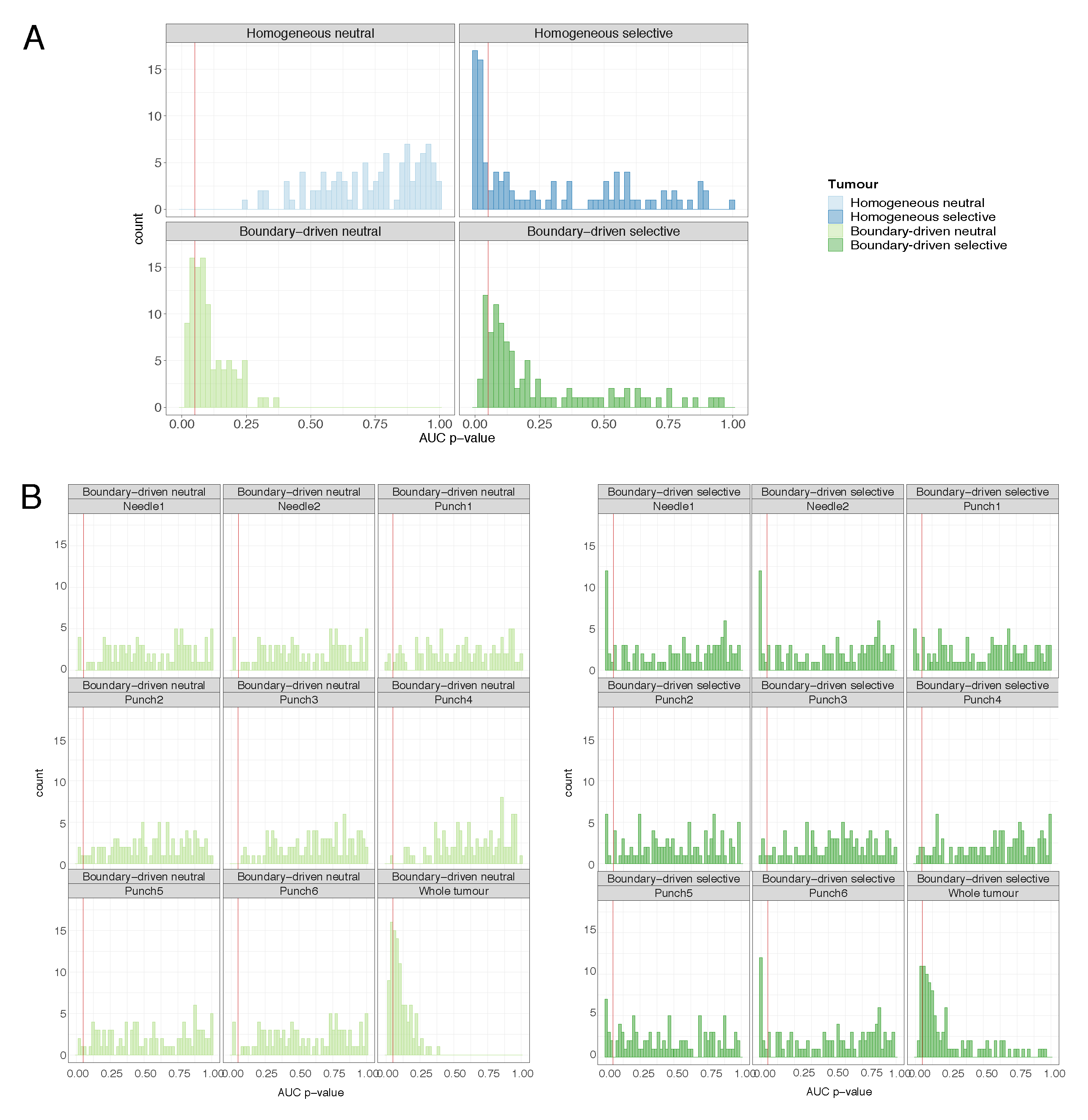

Supplement: S6 Fig — (A) We simulate 100 different tumours for each 4 representative growth models and fit 1/f test to their corresponding whole tumour sample VAFs. Reported are the distributions of p-values obtained from each test using the AUC statistics. (B) For the cases of boundary-driven growth modes we compared tests of neutrality using the whole-tumour sample versus punch/needle biopsies. (PNG) [file pcbi.1007243.s006.png]

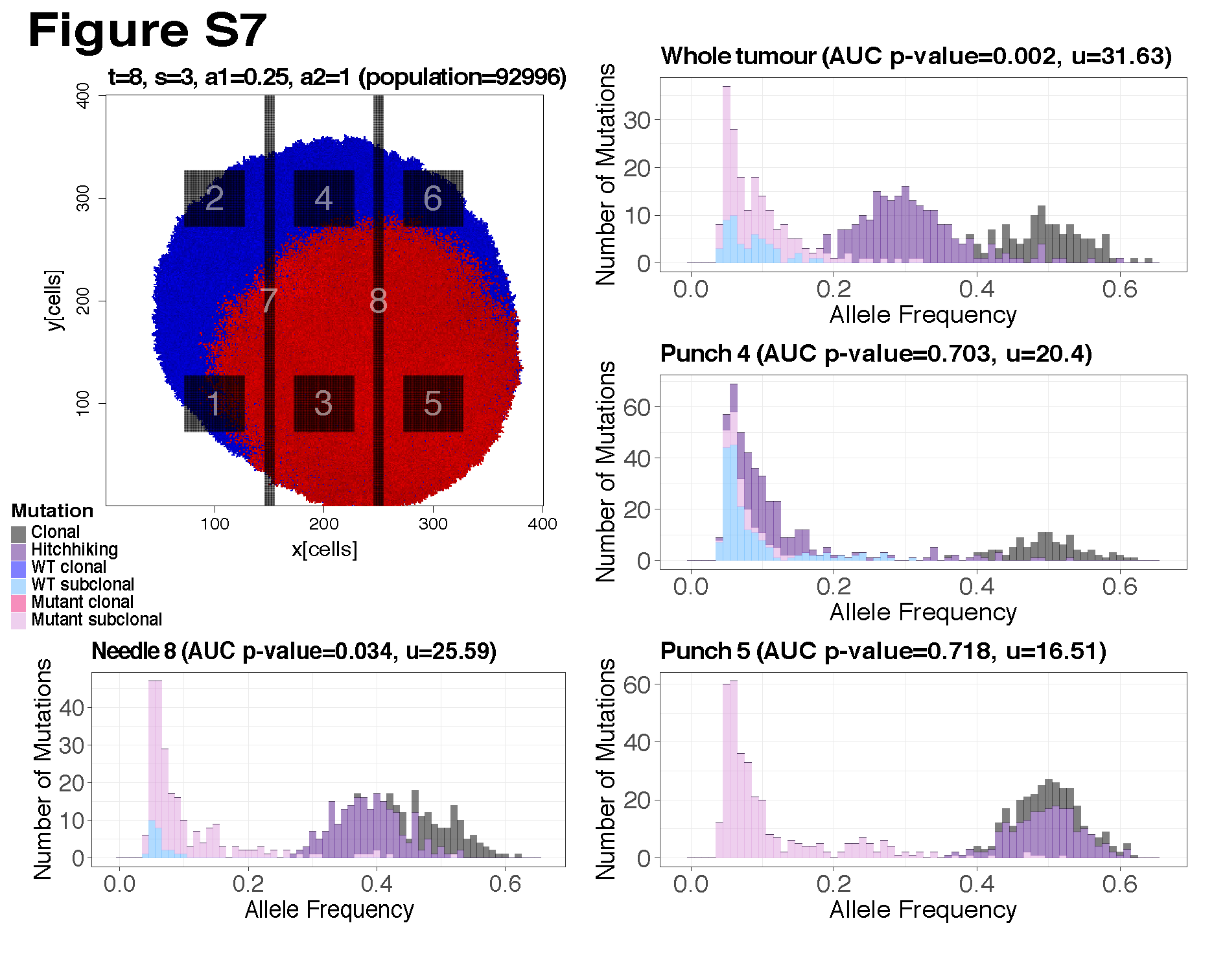

Supplement: S7 Fig — Example of a selective exponential growth when the mutant subpopulation has higher ‘push power’ than the wild type population. (PNG) [file pcbi.1007243.s007.png]

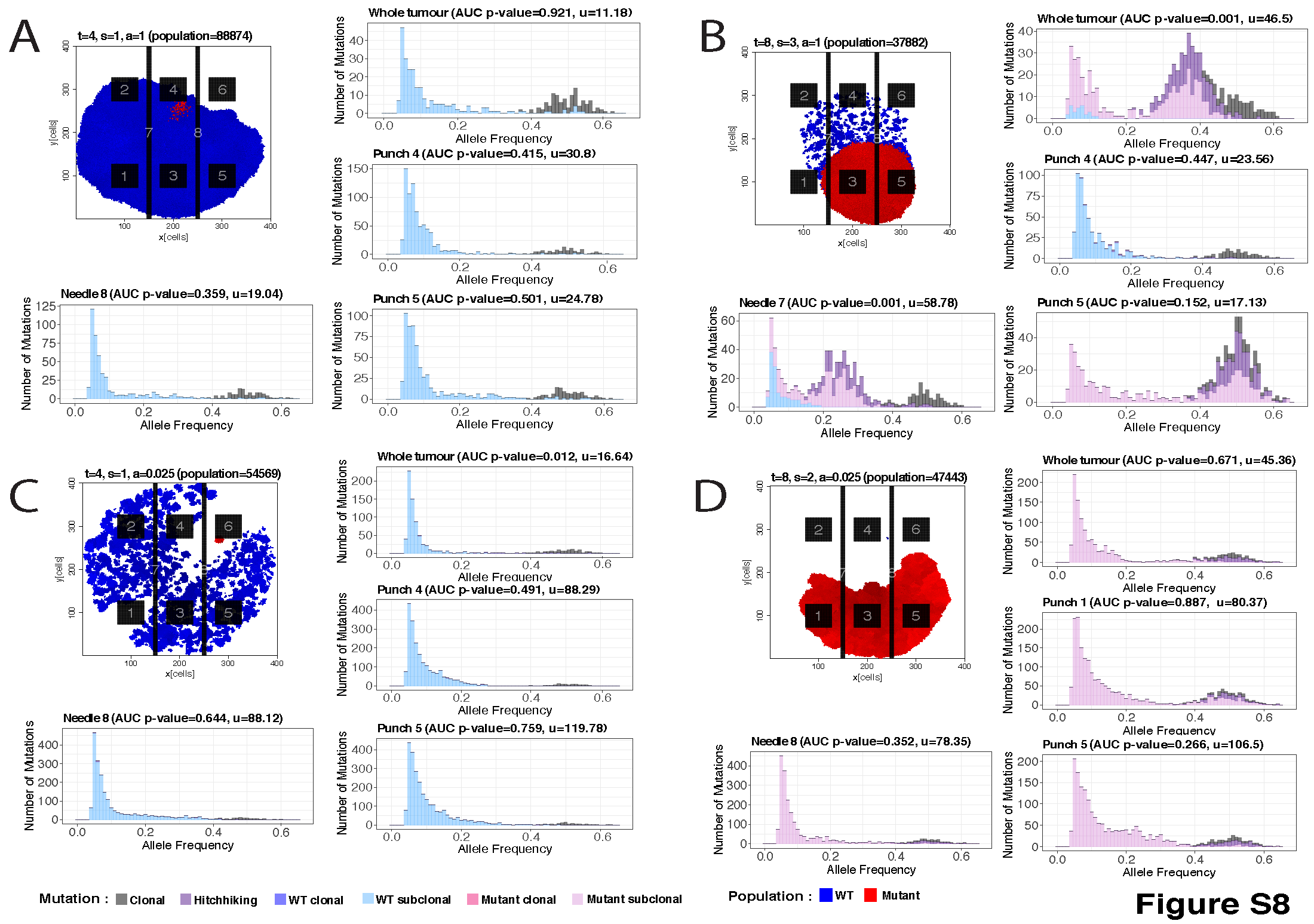

Supplement: S8 Fig — For each of the representative cases: (A) neutral homogeneous, (B) selective homogeneous, (C) neutral boundary driven, (D) selective boundary driven, we simulated procedures of removing large cell population (here 99%) by the end of tumour growth and wait for it to regrow to its original size. (PNG) [file pcbi.1007243.s008.png]

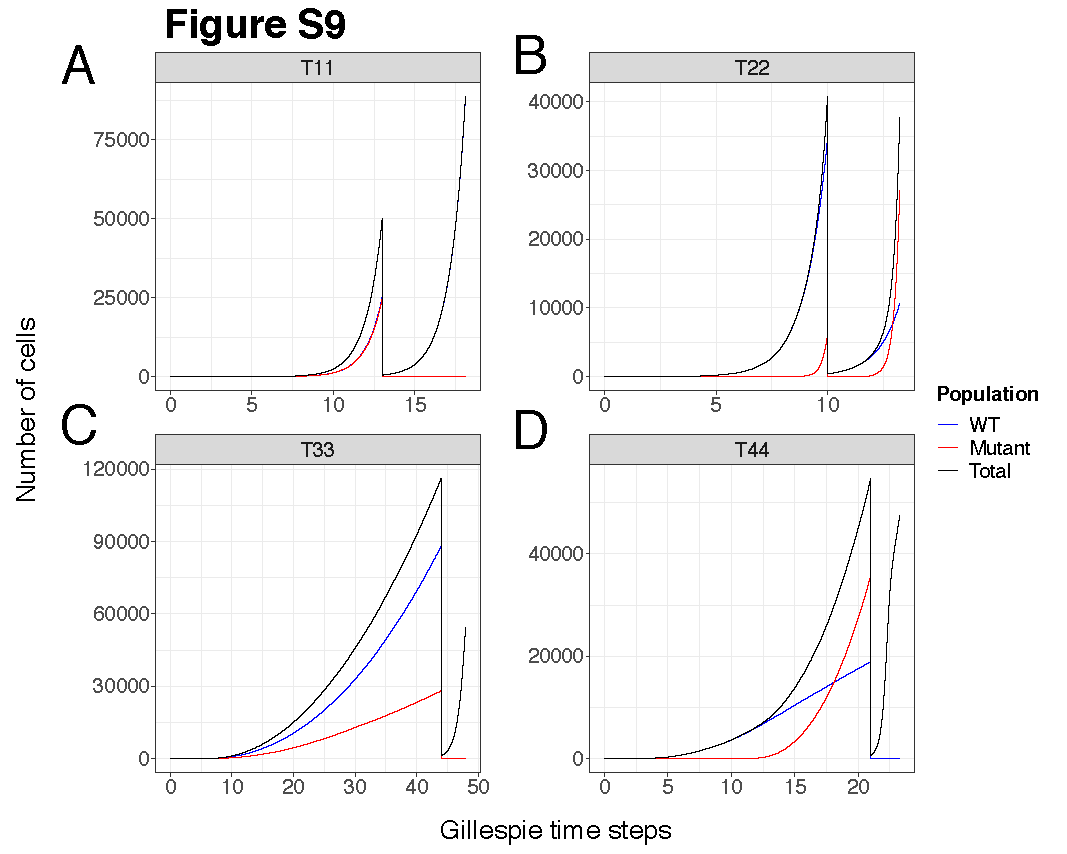

Supplement: S9 Fig — Tumour cell population growth curves for each of the representative cases: (A) neutral homogeneous, (B) selective homogeneous, (C) neutral boundary driven, (D) selective boundary driven, where by the end of tumour growth we remove 99% of the cell population and wait for the tumour to regrow to its original size. (PNG) [file pcbi.1007243.s009.png]

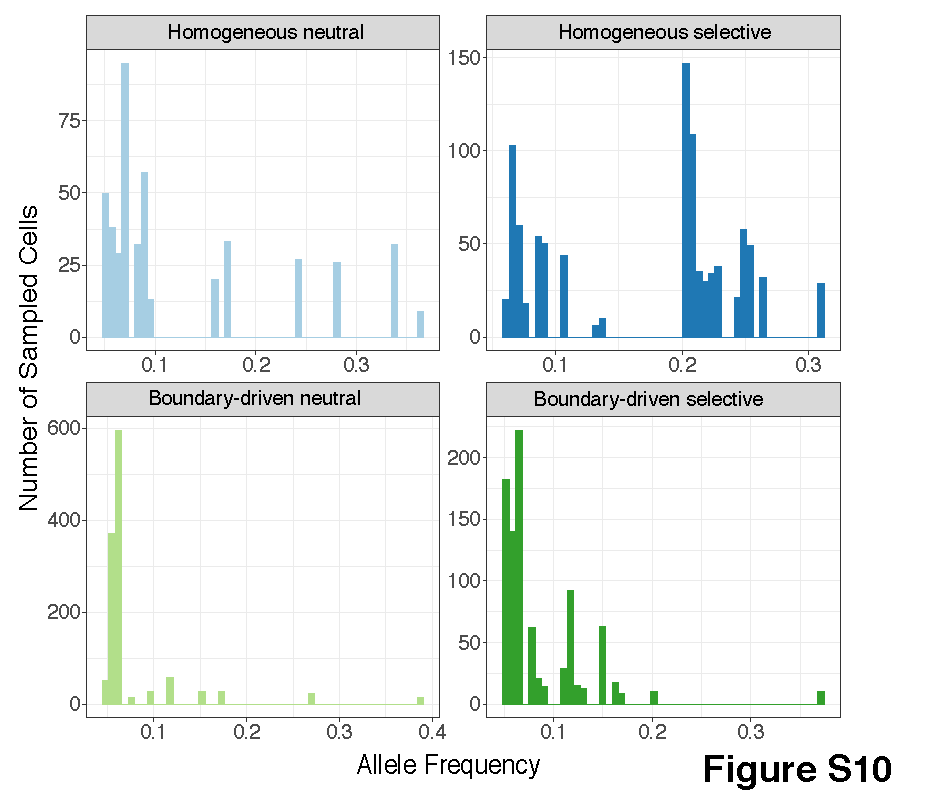

Supplement: S10 Fig — We construct the allele frequency distributions from sequencing the randomly sampled 400 single cells (same as in Fig 4) from the four representative tumour examples: T1 –neutral homogenous, T2 –selective homogenous, T3 –neutral boundary driven, T4 –selective boundary driven. (PNG) [file pcbi.1007243.s010.png]

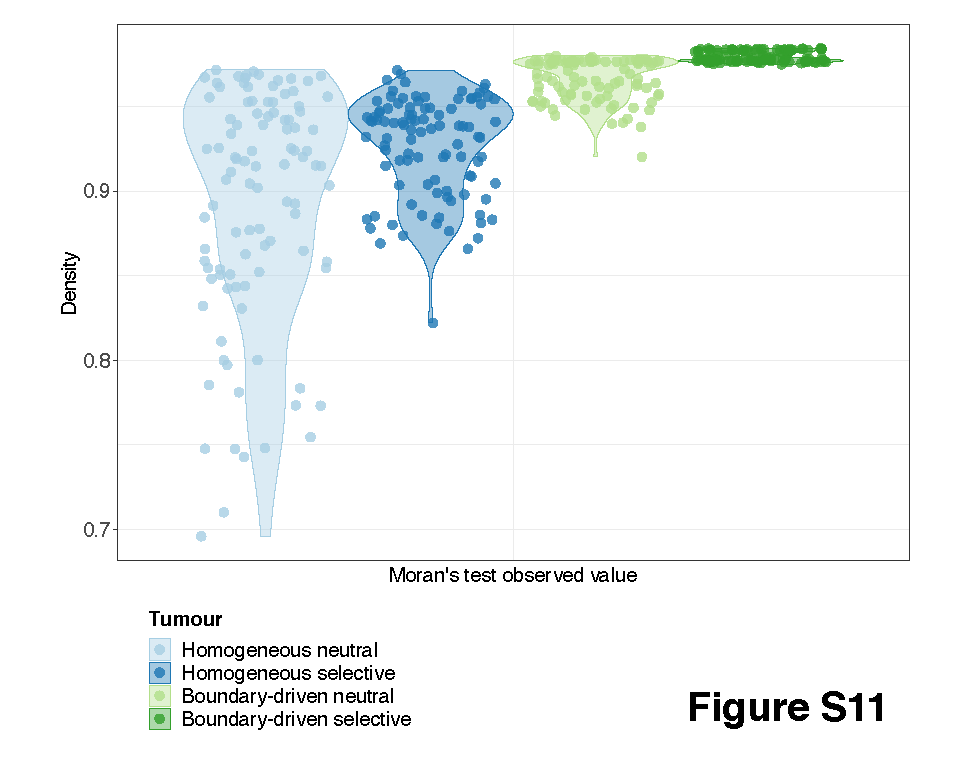

Supplement: S11 Fig — We simulate 100 different tumours for each 4 representative growth models and test intermixing of subpopulations within each simulation lattice using Moran’s entropy-based test. Each individual test output significant p-values indicating to high spatial correlation between tumour cell types (mutant vs WT) and their location on tumour lattice. Although the test effect size (the observed values of the Moran’s test statistic) differ as we can see from their distributions per model scenario. The median values of each observed statistics are reported at the bottom of each violin plot. (PNG) [file pcbi.1007243.s011.png]

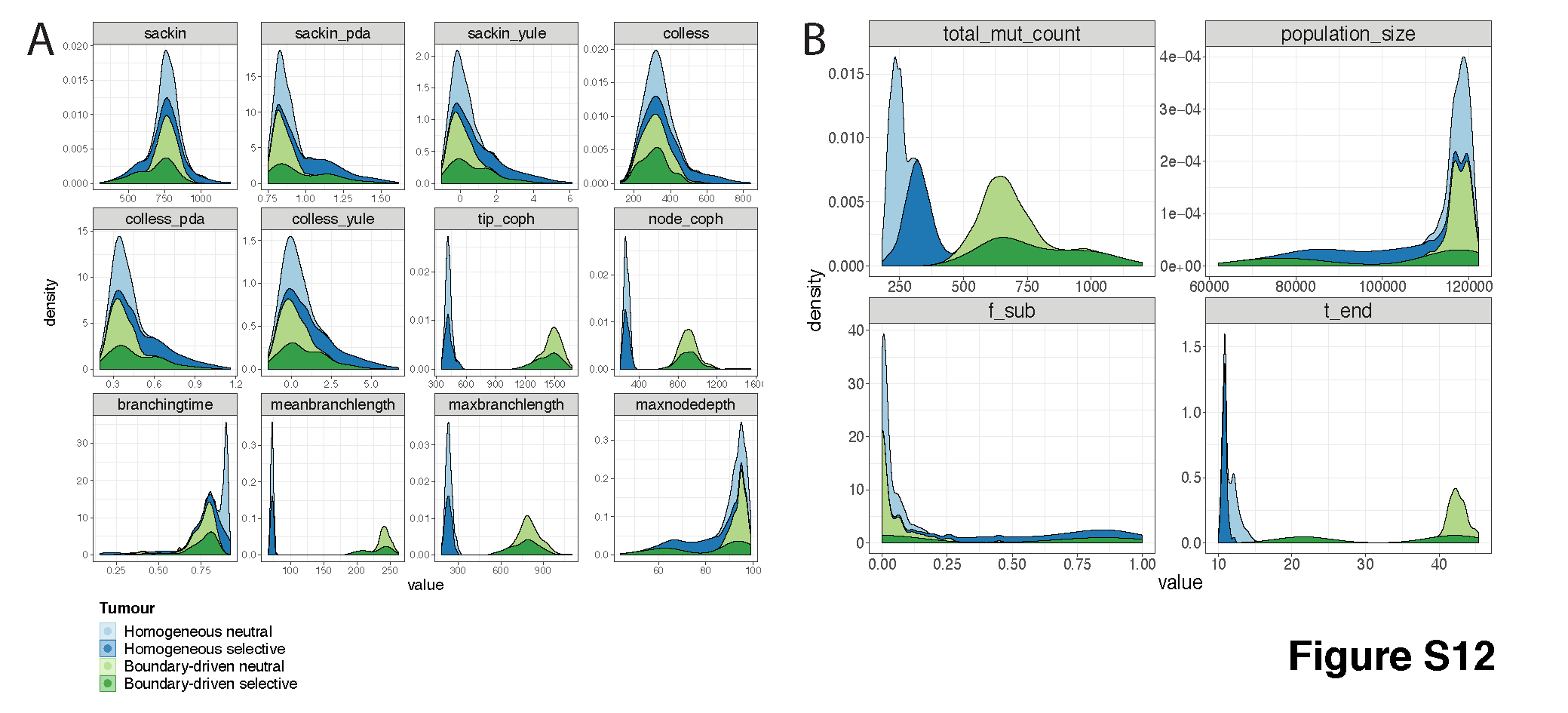

Supplement: S12 Fig — (A) Distributions of different summary statistics from single cell sampling (100x) phylogenetic trees for the four representative cases. The balance index-based statistics (sackin, colless with their different normalisation approaches–Yule, PDA) seem to have similar shapes among all four tumour cases, while tip and node Cophenetic distance-based statistics show different trends for neutral versus selective examples with not observable variation between homogenous and boundary driven tumours. Branch length-based statistics give similar results as cophenetic distances. Only one statistic, maximum node depth, tend to have longer flat tails for boundary driven tumours compared to homogenous tumour simulations. (B) For each of four tumour examples, we compare the total number of passenger mutations and final population sizes along with the time the simulations finish and the final frequency of the new sub-population (introduced after a driver event). (PNG) [file pcbi.1007243.s012.png]

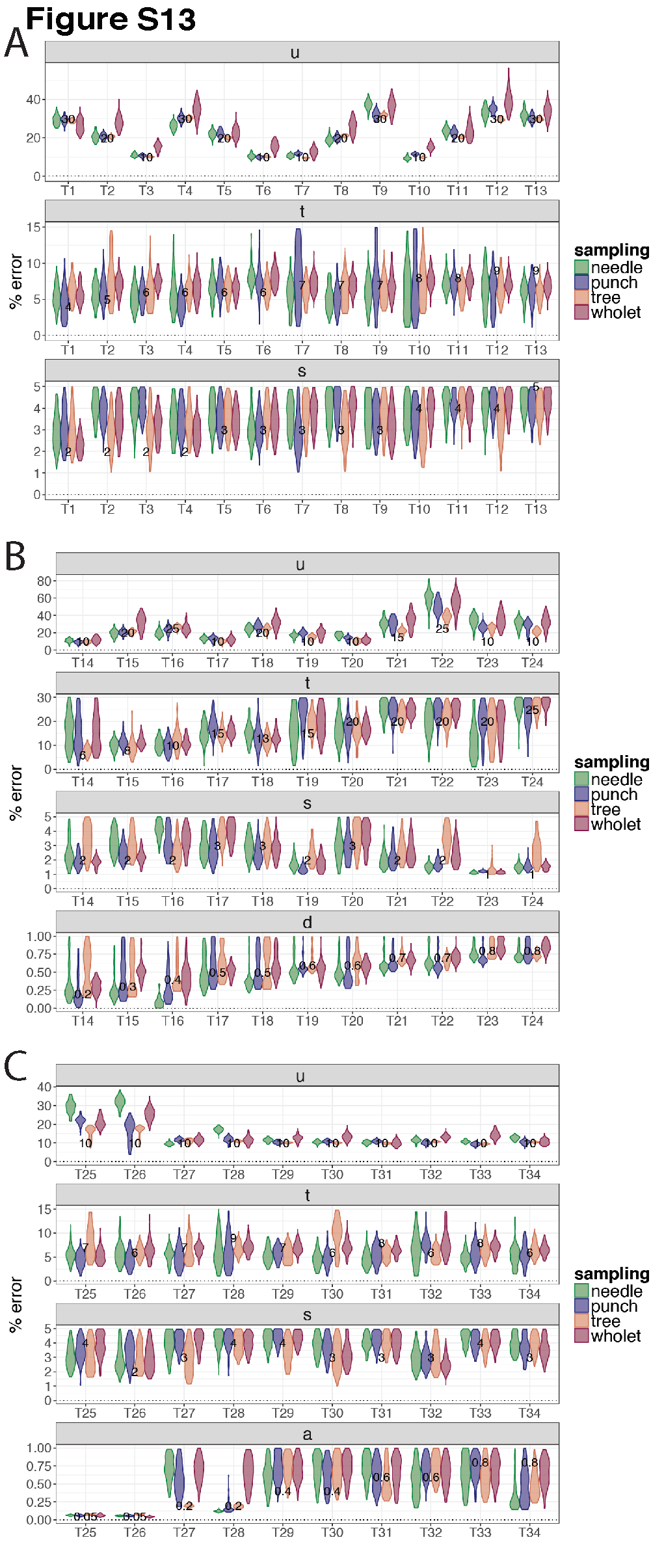

Supplement: S13 Fig — The violin plots of the posterior distributions for each model parameter per synthetic tumour inferred by our ABC-SMC framework. The three sets of tumours corresponding to the three tumour growth scenarios are plotted separately: exponential (A), death (B) and boundary driven (C). The number on the violin plots is the target value of each parameter. (PNG) [file pcbi.1007243.s013.png]

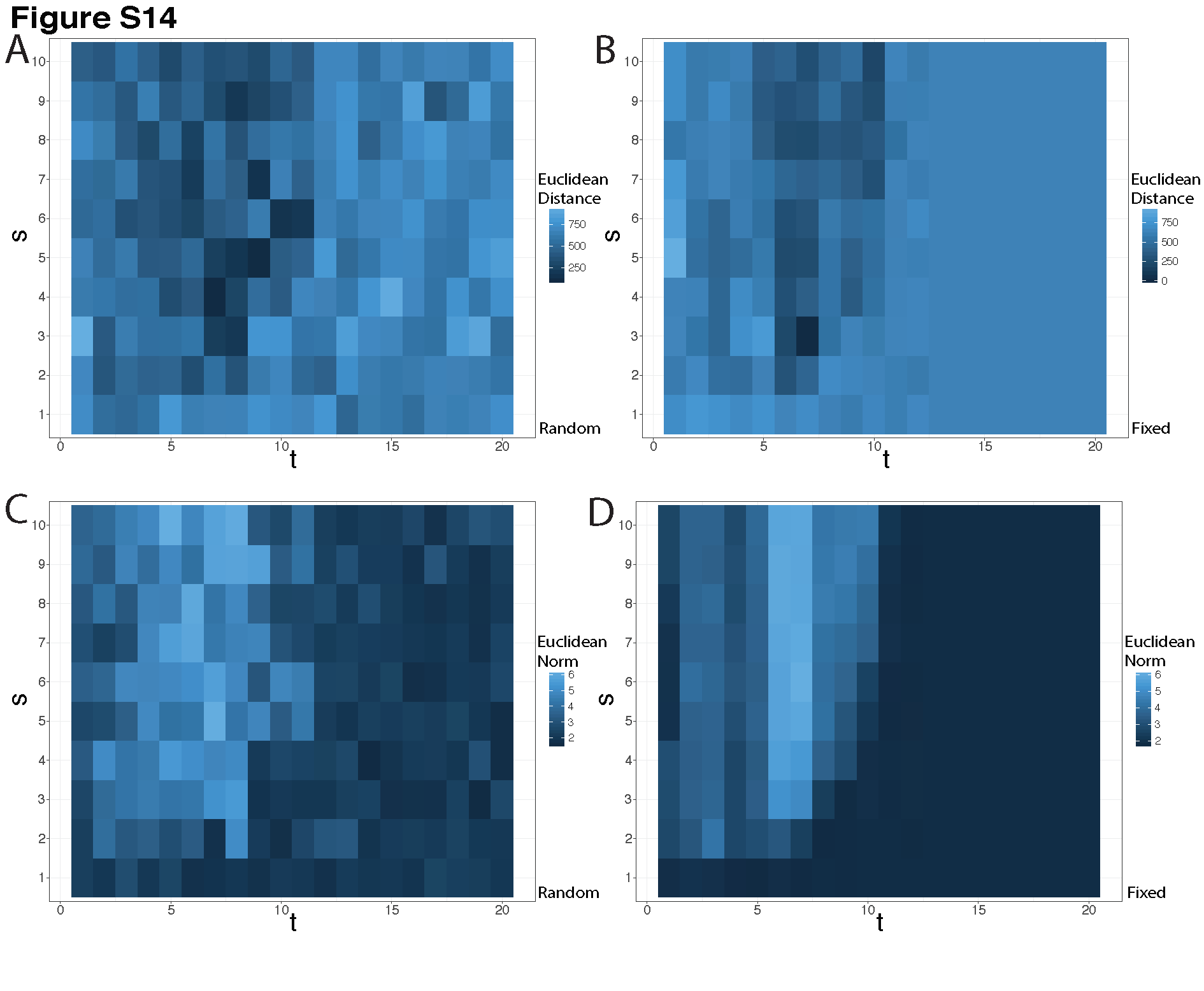

Supplement: S14 Fig — To explore the interdependence of the parameter pair t and s, for their different values we simulate tumour growth while fixing all the other parameters (2D grid size = 400, u = 10, d = 0, a = 1). We summarised the obtained tumours by calculating either the Euclidean norm of the obtained whole tumour VAFs (C, D) or the calculating Euclidean distance between the cumulative VAF distributions of the simulated and a chosen target tumour (in this case target tumour parameters are t = 7, s = 3) (A, B). To reduce the effect of stochasticity we fix the random seed in (B) and (D) and they indeed showed less scattered patterns of (A) and (C) plots respectively. (PNG) [file pcbi.1007243.s014.png]

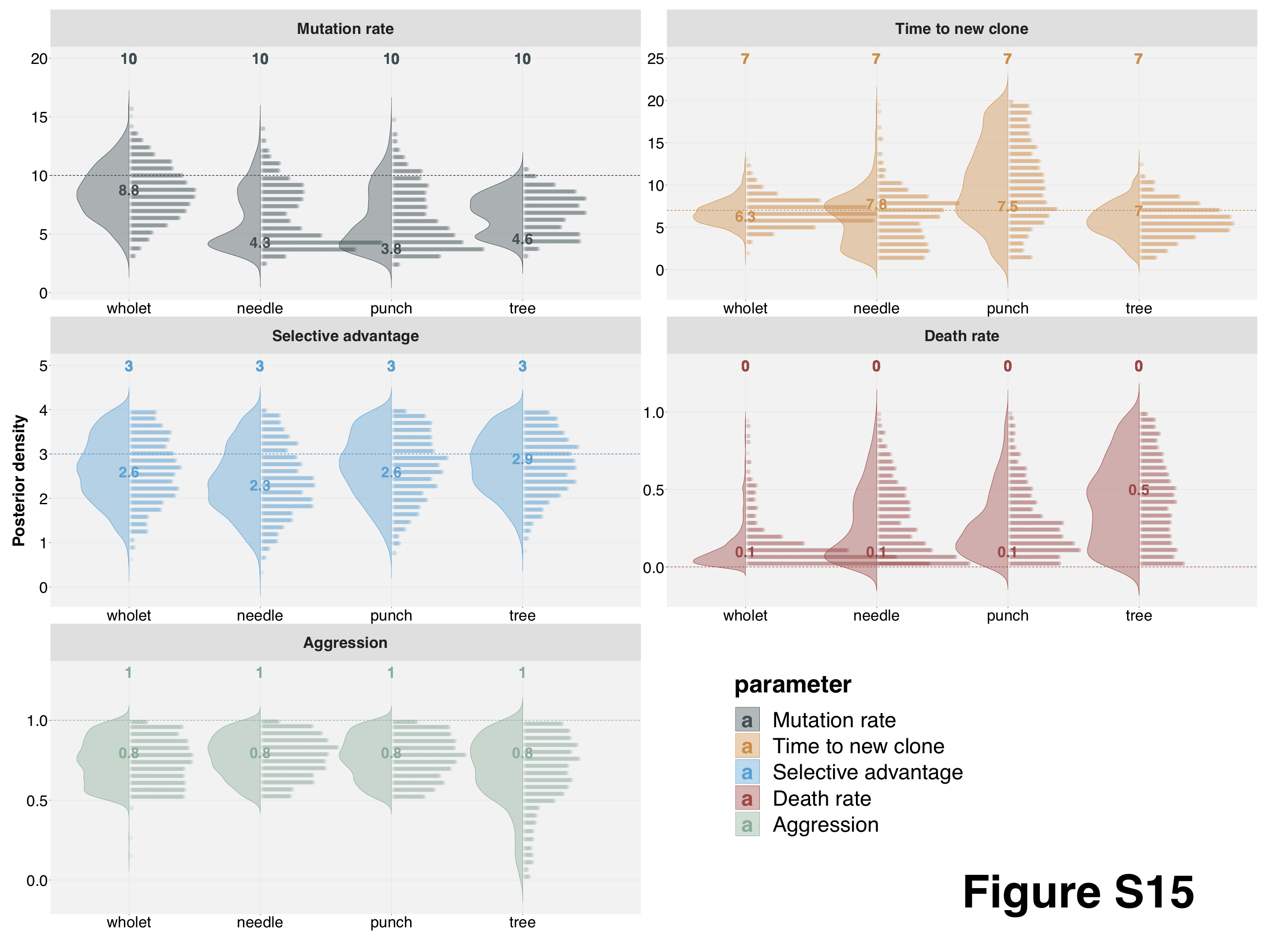

Supplement: S15 Fig — ABC SMC inference for a selective homogenous growth simulation in 3D space. Real ‘target’ values are reported as dashed lines. We run this ABC framework similarly to 2D simulations, where we recover each parameter at a time; first varying all parameters, once one is converged, fixing it at its inferred value and rerunning the simulation varying the parameters left to infer. Here we first recovered mutation rate, then time and selective advantage (together), and finally death rate and aggression (together as well). Similar to 2D models, our ABC framework with whole tumour sampling performs the best compared to other sampling strategies. (PNG) [file pcbi.1007243.s015.png]
